# Agrin Binds BMP2, BMP4 and TGFβ1

**DOI:** 10.1371/journal.pone.0010758

**Published:** 2010-05-21

**Authors:** László Bányai, Peter Sonderegger, László Patthy

**Affiliations:** 1 Institute of Enzymology, Biological Research Center, Hungarian Academy of Sciences, Budapest, Hungary; 2 Department of Biochemistry, University of Zurich, Zurich, Switzerland; University of Queensland, Australia

## Abstract

The C-terminal 95 kDa fragment of some isoforms of vertebrate agrins is sufficient to induce clustering of acetylcholine receptors but despite two decades of intense agrin research very little is known about the function of the other isoforms and the function of the larger, N-terminal part of agrins that is common to all isoforms. Since the N-terminal part of agrins contains several follistatin-domains, a domain type that is frequently implicated in binding TGFβs, we have explored the interaction of the N-terminal part of rat agrin (Agrin-Nterm) with members of the TGFβ family using surface plasmon resonance spectroscopy and reporter assays. Here we show that agrin binds BMP2, BMP4 and TGFβ1 with relatively high affinity, the K_D_ values of the interactions calculated from SPR experiments fall in the 10^−8^ M–10^−7^ M range. In reporter assays Agrin-Nterm inhibited the activities of BMP2 and BMP4, half maximal inhibition being achieved at ∼5×10^−7^ M. Paradoxically, in the case of TGFβ1 Agrin N-term caused a slight increase in activity in reporter assays. Our finding that agrin binds members of the TGFβ family may have important implications for the role of these growth factors in the regulation of synaptogenesis as well as for the role of agrin isoforms that are unable to induce clustering of acetylcholine receptors. We suggest that binding of these TGFβ family members to agrin may have a dual function: agrin may serve as a reservoir for these growth factors and may also inhibit their growth promoting activity. Based on analysis of the evolutionary history of agrin we suggest that agrin's growth factor binding function is more ancient than its involvement in acetylcholine receptor clustering.

## Introduction

**Figure 1:**
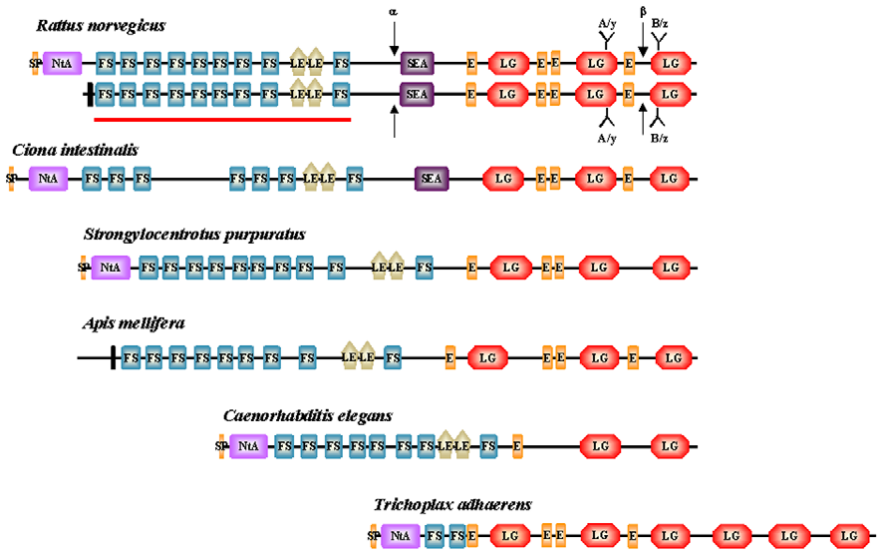
Domain architectures of agrins. Different domain-types of agrins are: N-terminal agrin domain, NtA (NtA; light purple); Follistatin-domains, (FS; blue), laminin-EGF domains (LE; light brown), SEA-domain (SEA; purple); EGF-domains (E; orange), laminin G domains (LG; light red). Vertebrate agrins (represented here by *Rattus norvegicus* agrin) have two alternative N-termini. The secreted form has a signal sequence (SP; orange) and a laminin-binding N-terminal agrin domain, whereas the membrane-bound form has a short intracellular region and a transmembrane helix (vertical black bar). Alternative splicing in the C-terminal part of vertebrate agrins at B/z site gives rise to agrin isoforms that differ in their ability to cluster acetylcholin receptors. The two arrows indicate the neurotrypsin cleavage sites (α and β sites) of vertebrate agrins. Note that the neurotrypsin cleavage sites are not conserved in invertebrate agrins. The solid red line indicates the domain structure of recombinant Agrin_Nterm used in the present study. The lower part of the figure shows the structures of agrins of invertebrate species representing Urochordates (*Ciona intestinalis*), Echonoderms (*Strongylocentrotus purpuratus*), Arthropods (*Apis mellifera*), Nematodes (*Caenorhabditis elegans*) and Placozoa (*Trichoplax adhaerens*); for details see TableS1. Note that agrin of the Arthropod *Tribolium castaneum* has the same domain architecture as agrin of *Apis mellifera*.

The proteoglycan agrin is crucial for development and maintenance of the neuromuscular junction (NMJ) in vertebrates [1], [2] and it may also have synapse-promoting functions in the CNS [3]–[8]. Vertebrate agrins exist in several isoforms that are generated by alternative splicing (Figure 1). Differential transcription of the first exon of the agrin gene results either in a secreted form that binds to the basal lamina via its laminin-binding N-terminal NtA domain or a shorter isoform without the NtA domain that remains attached to the cellular plasma membrane as a type-II transmembrane protein. The type II transmembrane isoform of agrin predominates in the brain, whereas the secreted variant is the predominant form expressed by motoneurons. Secreted agrin is released from the nerve ending of motoneurons into the synaptic cleft of the neuromuscular junction, where it becomes an essential component of the synaptic basal lamina.

The N-terminal part of all forms of vertebrate agrins consist of nine follistatin-related and two laminin EGF-like modules [9], the middle part contains a SEA module [10], and the C-terminal part contains four epidermal growth factor and three laminin globular domains (Figure 1). With the C-terminal laminin G domain motoneuron-derived agrin affects NMJ formation and maintenance by binding to a receptor complex in the muscle membrane consisting of MuSK (muscle-specific kinase) and LRP4 (low-density lipoprotein receptor-related protein 4)[11], [12].

In vertebrates, alternative splicing at a conserved site in the C-terminal part (Figure 1) gives rise to agrin isoforms with significantly different activities in clustering acetylcholine receptors (AChRs). The isoforms expressed by motoneurons contain an insert of 8, 11, or 19 amino acids at this site and are active in AChR clustering, whereas agrin expressed by muscle has no insert at this site and does not cluster AChRs [1], [13]. Recently, the C-terminal Laminin G domain of agrin was found to be instrumental for the activity-dependent promotion of dendritic filopodia on hippocampal neurons, after its activity-dependent proteolytic release from its parent agrin by the neuronal protease neurotrypsin [6]. In contrast with NMJ formation, activity-dependent promotion of dendritic filopodia does not require an insert at the B/z splice site present in this Laminin G domain [6].

Little is known about the function of agrin's N-terminal region. Based on homology with follistatin, we have suggested previously that this region, common to all agrin isoforms, might bind growth factors of the TGFβ family [9]. In the present study we produced and purified a recombinant N-terminal fragment of agrin (Agrin-Nterm) and used it to study its interaction with various members of the TGFβ family with surface plasmon resonance and reporter assays. SPR studies have revealed that Agrin-Nterm has relatively high affinity for BMP2, BMP4 and TGFβ1 and in reporter assays it inhibits the activity of BMP2 and BMP4 but enhances the activity of TGFβ1. Our finding that Agrin-Nterm binds members of the TGFβ family may have important implications for the role of these growth factors in the regulation of synaptogenesis as well as for the role of agrin isoforms that are unable to induce clustering of acetylcholine receptors.

## Materials and Methods

### Reagents, enzymes, PCR primers, proteins, bacterial strains, cell lines and media

Restriction enzymes, T4 DNA Ligase and Klenow polymerase were New England Biolabs products (Beverly, MA, USA). PCR primers were obtained from Integrated DNA Technologies (Coralville, IA, USA). For amplification reactions we used Taq polymerase from Fermentas (Vilnius, Lithuania) or the proof-reading thermostable polymerase Accuzyme (Bioline, London, UK). DNA purification was performed with Nucleospin Extract PCR purification kit (Macherey-Nagel, Duren, Germany). *Escherichia coli* JM109 bacterial strain was used for DNA propagation during DNA manipulation steps. Mature human BMP2, BMP4, TGFβ1 and TGF-βsRII (corresponding to the Extracellular domain of TGF-βRII) were purchased from R&D Systems (Wiesbaden, Germany). CM5 sensorchips and the reagents for protein coupling to the chips were from Biacore AB (Uppsala, Sweden). The extracellular domain of human BMPR1A (ECD-BMPR1A) was produced as described in a separate publication (Szláma, Kondás, Trexler and Patthy, manuscript submitted to JBC).

The firefly luciferase kit was from Biotium (Hayward, CA, USA). Mink lung epithelial cells stably transfected with a truncated PAI-1 promoter/firefly luciferase construct (MLEC-clone32) [14] and HepG2-BRA cells stably transfected with the BRE-luc reporter construct [15] were generously provided by Professor Daniel Rifkin (New York University). Culture media DMEM and heat inactivated FBS were obtained from Sigma-Aldrich (St Louis, Mo USA).

### Cloning and expression of the N-terminal region of rat agrin

The cDNA segment encoding residues Asp65-Gln865 of rat agrin (Agrin-Nterm) was amplified with the 5′-GCAGATCTGATGTATGCAGGGGAATGTTATGTGG -3′ sense and 5′-GCTCTAGACTGGCAGGGACCAAGACTCTG-3′ antisense primers using rat agrin cDNA (NCBI Reference Sequence: NM_175754.1).

The amplified DNA was digested with BglII and XbaI restriction endonucleases and ligated into *Drosophila* expression vector pMT/BiP/V5-His A digested with the same enzymes. The ligation mixture was transformed into *Escherichia coli* JM109 cells and the recombinants were selected on LB medium with 100 mg/ml Ampicillin. Plasmids from transformants were isolated and analysed for the presence of insert. The sequence of the cloned DNA was verified on both strands.

*Drosophila melanogaster* S2 cells (DGRC Indiana University, Bloomington, IN USA) were transfected with 4 µg pMT/BiP/V5-His A expression plasmid containing the cDNA encoding Agrin-Nterm and 16 µg pCoHygro selection vector using Cellfectin reagent (Invitrogen, Carlsbad, CA USA) according to the protocol recommended by the manufacturer. For selection of stable transfectants, cells were suspended and cultured in Schneider's *Drosophila* medium (Invitrogen, Carlsbad, CA USA) supplemented with 10% Fetal bovine serum (Sigma-Aldrich,) and 300 µg/ml hygromycin B (Invitrogen, Carlsbad, CA USA). Stably transformed polyclonal lines were established after 5 weeks of selection with hygromycin B. Except for propagation in serum-free medium hygromycin B was always included in the media.

For protein induction stable transfectants were grown in serum free medium (Invitrogen, Carlsbad, CA USA) to a cell density of 2–3×10^6^/ml and protein expression was induced by adding CuSO_4_ at 400 µmol final concentration. After 1 week of induction the culture was centrifuged, the conditioned medium was harvested and the cells were suspended in fresh induction medium to start another round of induction. Usually three rounds of induction were performed with the same cells. The medium collected from three rounds of induction was dialyzed against 25 mM Tris pH 7.5 buffer.

### Purification of Agrin-Nterm

**Figure 2:**
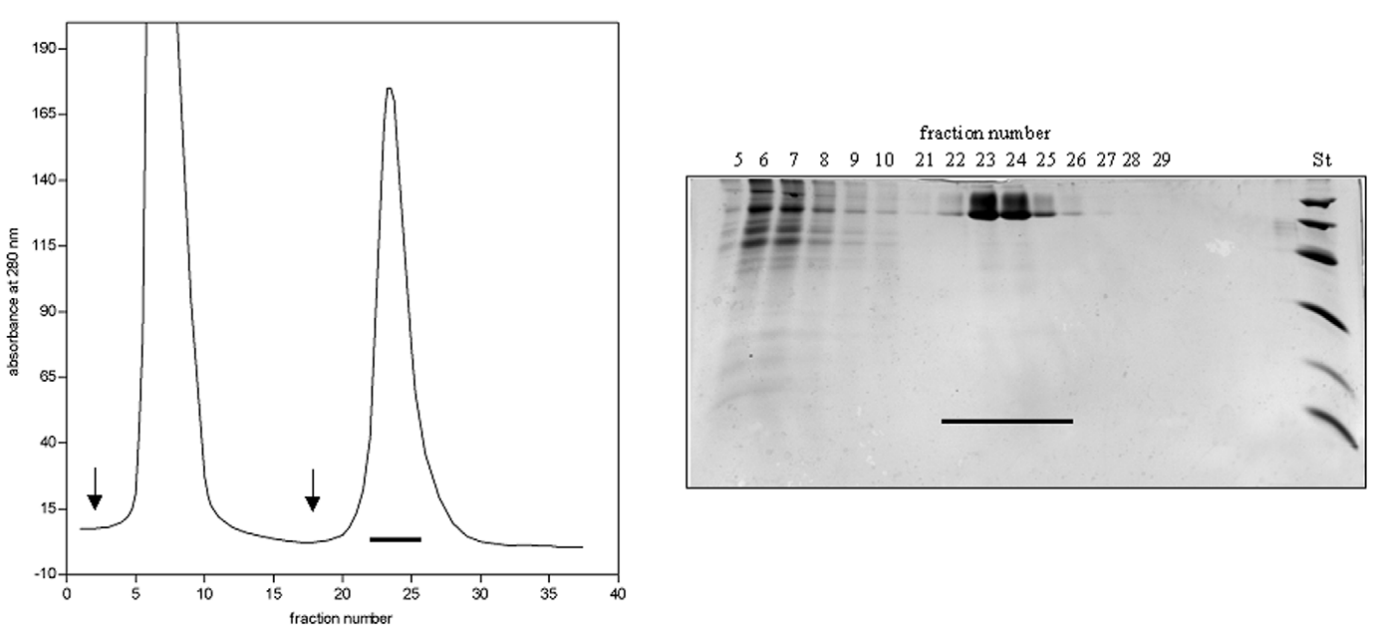
Isolation of Agrin-Nterm by affinity chromatography. Dialyzed culture fluid of *Drosophila melanogaster* S2 cells expressing recombinant Agrin_Nterm was applied onto a Ni affinity column, the column was washed with 10 column volumes of 20 mM Tris-HCl buffer, pH 7.9 containing 500 mM NaCl and 5 mM imidazole, then with 5 column volumes of 20 mM Tris-HCl buffer, pH 7.9 containing 500 mM NaCl and 30 mM imidazole (first arrow) and the bound protein was eluted with 20 mM Tris-HCl buffer, pH 7.9 containing 300 mM imidazole (second arrow). The right panel shows the SDS-PAGE of the affinity chromatography, St indicates the pattern of the Low Molecular Weight Calibration Kit (Amersham Pharmacia Biotech, Uppsala, Sweden; Mr values: 14,400, 20,100, 30,000, 45,000, 66,000, and 97,000). The horizontal bars indicate the pooled fractions containing Agrin-Nterm.

**Figure 3:**
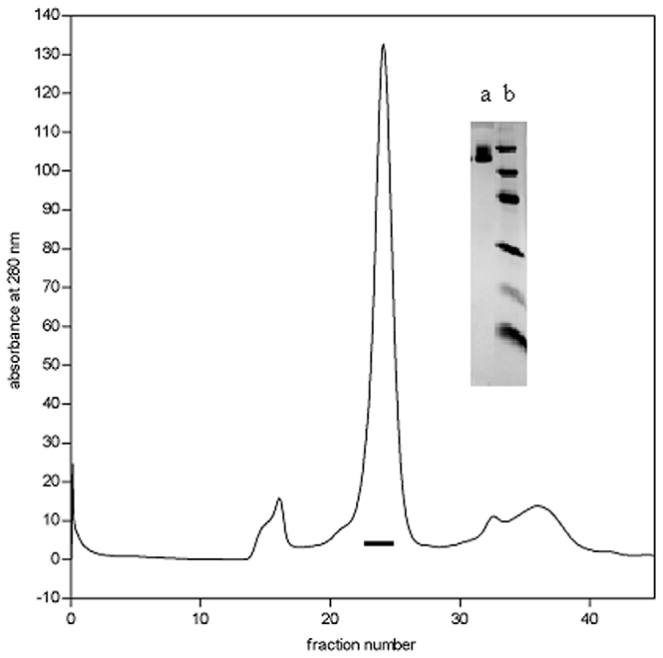
Purification of Agrin-Nterm by gel chromatography. Recombinant protein isolated by Ni affinity chromatography (seeFig. 2.) was chromatographed on a AKTA purifier 10 System using a Superdex gel filtration column (HiLoad 10/300 Superdex 200 GL) and fractions containing pure Agrin-Nterm (horizontal bar) were pooled. Lane a of the insert shows SDS-PAGE of Agrin-Nterm of the pooled factions of AKTA chromatography, lane b indicates the pattern of the Low Molecular Weight Calibration Kit (Amersham Pharmacia Biotech, Uppsala, Sweden; Mr values: 14,400, 20,100, 30,000, 45,000, 66,000, and 97,000).

Dialyzed culture fluid was applied onto a Ni affinity column (Amersham Biosciences UK). The column was washed with 10 column volumes of 20 mM Tris-HCl buffer, pH 7.9 containing 500 mM NaCl and 5 mM imidazole, then with 5 column volumes of 20 mM Tris-HCl buffer, pH 7.9 containing 500 mM NaCl and 30 mM imidazole and the bound protein was eluted with 20 mM Tris-HCl buffer, pH 7.9 containing 300 mM imidazole (Figure 2). The eluted protein was desalted on a Sephadex G-25 column equilibrated with 100 mM ammonium bicarbonate, pH 8.0 buffer and lyophilized. Recombinant protein was further purified with AKTA purifier 10 System (GE Healthcare, Uppsala, Sweden) using a Superdex gel filtration column (HiLoad 10/300 Superdex 200 GL) equilibrated with 100 mM ammonium bicarbonate buffer, pH 8.0 (Figure 3). Fractions of 0.5 ml were collected, samples were analysed by SDS–PAGE and the fractions containing pure Agrin-Nterm were pooled and lyophilized. The average yield of purified protein was 1.0–1.5 mg per liter tissue culture fluid.

### Protein analyses

The composition of protein samples was analysed by SDS–PAGE using 6–16% linear polyacrylamide gradient slab gels under both reducing and non-reducing conditions. The gels were stained with Coomassie Brilliant Blue G-250. On SDS-PAGE recombinant Agrin-Nterm appeared as a broad, high molecular smear (M_r_>90 kDa) characteristic of proteoglycans. The calculated molecular mass of recombinant Agrin-Nterm is 88,054 Da; the difference of predicted and observed molecular mass of Agrin-Nterm is due to glycosylation at multiple sites in this part of agrin [16].

The concentration of recombinant protein was determined using the extinction coefficient of 45475 M−1 cm−1. The extinction coefficient was determined with ExPASy's ProtParam tool (http://us.expasy.org/tools/protparam.html).

N-terminal sequencing was performed on an Applied Biosystems 471A protein sequencer with an online ABI120A PTH Amino Acid Analyser. The N-terminal sequence of Agrin-Nterm was RSDVCRGMLCGF (the residues in bold underline correspond to residues 65–74 of rat agrin).

### Surface plasmon resonance analysis

Surface plasmon resonance measurements were performed on a BIAcore X instrument (GE Healthcare, Stockholm, Sweden) essentially as described previously [17].

Recombinant human proteins (BMP2, BMP4, TGFβ1, TGFβ-sRII) were dissolved according to the instructions of the manufacturer (R&D Systems, Wiesbaden, Germany). The proteins were diluted in 50 mM sodium acetate buffer, pH 4.5 at a final concentration of 0.04 µg/ml (TGFβ1 and BMPs) and 50 µl of these solutions were injected with a 5 µl/min flow rate for 10 min on a CM5 sensor chip (Biacore AB, Uppsala, Sweden) activated by the amine coupling method according to the instructions of the manufacturer. ECD-BMPRIA-sensor chips were prepared in a similar way, except that the protein was dissolved in 50 mM sodium acetate, pH 4.2 ((Szláma, Kondás, Trexler and Patthy, manuscript submitted to JBC). For interaction measurements, 70 µl samples containing different concentrations of the analyte were injected on the sensor-chips at a flow rate of 20 µl/min, followed by wash with buffer at a flow rate of 20 µl/min.

Binding and washes were performed in 20 mM HEPES, 150 mM NaCl, 5 mM EDTA, 0.005% Tween 20, pH 7.5 buffer. Regeneration of the chip surface after each cycle was performed with injection of 40 µl 20 mM HEPES, 150 mM NaCl, 5 mM EDTA, 0.005% Tween 20, pH 7.5 buffer containing 8 M urea over the sensor chip. All experiments were repeated at least twice. Reference cells were used to obtain control sensorgrams showing non-specific binding to the surface as well as refractive index changes resulting from changes in bulk properties of the solution. Reference flow cells were prepared by executing the coupling reaction in the presence of coupling buffer alone. Reference sensorgrams were subtracted from sensorgrams obtained with immobilized ligand. To correct for differences between the reaction and reference surfaces we have also subtracted the average of sensorgrams obtained with blank running buffer injections.

The kinetic parameters (k_a_ – association rate constant; k_d_ – dissociation rate constant; K_D_ = k_d_/k_a_ – equilibrium dissociation constant) for each interaction were determined by globally fitting the experimental data with BIAevaluation software 4.1 and the closeness of the fits was characterized by the χ^2^ values. Only fits with χ^2^ values lower than 5% of the Rmax were accepted. Data were fitted to a model of 1∶1 Langmuir interaction.

In solution-competition assays, constant concentrations of growth factors were incubated with increasing concentrations of Agrin-Nterm in 20 mM HEPES, 150 mM NaCl, 5 mM EDTA, 0.005% Tween 20 pH 7.5 buffer for 30 min at room temperature prior to injection on chips with immobilized ECDs of growth factor receptors.

### Cell culture

Mink lung epithelial cells and HepG2-BRA cells were cultured in DMEM supplemented with 10% FBS, penicillin (100 U/ml) streptomycin (100 µg/ml) and geneticin at a concentration of 200 µg/ml (MLEC-clone32) or 700 µg/ml (HEPG2-BRA) at 37°C, 5%CO_2_.

### Reporter assays

TGFβ1 activity was measured with MLEC-clone32 cells, whereas the activities of BMP2 and BMP4 were monitored with HEPG2-BRA cells, using 96-well tissue culture dishes. In these reporter assays MLEC-clone32 cells (1.6×10^4^ cells/well) or HEPG2-BRA cells (5×10^3^ cells/well) were allowed to attach for 3 hours or 24 hours respectively, then the medium was changed to DMEM supplemented with 0.1% BSA, penicillin (100 U/ml) streptomycin (100 µg/ml) containing 16 pM TGFβ1, 250 pM BMP2 or 250 pM BMP4 preincubated for 30 minutes with different concentrations of Agrin-Nterm. Control experiments were performed similarly, except that no growth factor was added.

After incubation for 17 hours at 37°C, 5%CO_2_ the cells were lysed in 100 µl lysis buffer and the luciferase activity of the samples were determined using the firefly luciferase assay kit of Biotium on an Appliskan luminometer (Thermo Electron Corporation, MA, USA). The protein content of the samples was determined with the Bio-Rad protein assay (Biorad, USA) and the luciferase activity was normalized to the protein content of the wells.

### Sequence retrieval and analysis

Protein sequences of vertebrate agrins were retrieved from the NCBI (http://www.ncbi.nlm.nih.gov/sites/entrez) and UniProt (http://www.uniprot.org/) websites and were used as queries in BLAST searches (http://blast.ncbi.nlm.nih.gov/Blast.cgi) of protein, nucleotide and genomic databases to identify agrin orthologs of invertebrate species. In these analyses we focused on species with completely sequenced genomes representing major groups of Metazoa: the Placozoan *Trichoplax adhaerens* (http://genome.jgi-psf.org/Triad1/Triad1.home.html), the Nematodes *Caenorhabditis elegans* and *Caenorhabditis elegans*, the Arthropods *Drosophila melanogaster, Drosophila pseudoobscura, Apis mellifera*, *Tribolium castaneum*), the Echinoderm *Strongylocentrotus purpuratus* and the Urochordate *Ciona intestinalis*.

Invertebrate proteins identified in these searches were considered to be orthologs of vertebrate agrins if they satisfied the following criteria: 1) in reciprocal searches of vertebrate sections of protein sequence databases they gave the lowest E-scores with agrins; 2) in reciprocal searches of vertebrate sections of protein sequence databases the individual constituent domains of the candidate sequences gave the lowest E-scores with agrins.

Accordingly, appropriate invertebrate taxonomic sections of databases were first queried with vertebrate agrin sequences to identify proteins that gave the lowest E-scores then the ten top-scoring sequences were used in reciprocal searches of vertebrate sections of protein sequence databases. Sequences that gave the lowest E-scores with agrins were subjected to protein domain analyses using Pfam 23.0 (http://pfam.sanger.ac.uk/) and PfamA domains were identified with the search strategy ‘global and local (merged)’ using an E-value cut-off of 1.0. Sequences that contained NtA-, follistatin-, laminin EGF-, SEA or laminin G-domains were retained for further analysis (note that in Pfam 23.0 follistatin domains are identified as Kazal_1 or Kazal_2 domains).

In the final step individual domains of candidate agrin sequences were used as query to test whether in BLAST searches of vertebrate sections of sequence databases they give the lowest E-scores with agrins. Invertebrate sequences that passed this test were considered to be true orthologs of vertebrate agrins.

Invertebrate agrin sequences were analysed by the Mispred Procedure [18], [19] to identify and correct possible errors in gene prediction. To correct errors of predicted sequences, the genomic regions encoding the candidate agrin sequences were reanalysed by GENSCAN (http://genes.mit.edu/GENSCAN.html), GenomeScan (http://genes.mit.edu/genomescan.html), Augustus (http://augustus.gobics.de/), Wise2 (http://www.ebi.ac.uk/Tools/Wise2/index.html) and predictions that corrected the error(s) identified by MisPred were selected.

The domain architectures of invertebrate agrins were compared with those of vertebrate agrins and the validity of deviations (e.g. presence/absence of domains) was checked using the consensus sequence procedure [20]. Regions with less significant PfamA matches (10^−5^<E value<10^−2^) or regions (>100 residues) with no significant PfamA hits were analyzed with the consensus sequence procedure [20] to decide whether they are significantly related to known domain families.

The results of these analyses are summarized in Table S1 and Figures S1, S2, S3, S4, S5, S6. Multiple alignments of the sequences of constituent domains of agrins were obtained with ClustalW [21]. The multiple alignments were shaded using Boxshade 3.0. Identical residues conserved in the majority of sequences were highlighted with black bakground, chemically similar residues were highlighted with grey background.

## Results

### Agrin binds BMP2, BMP4 and TGFβ1

**Figure 4:**
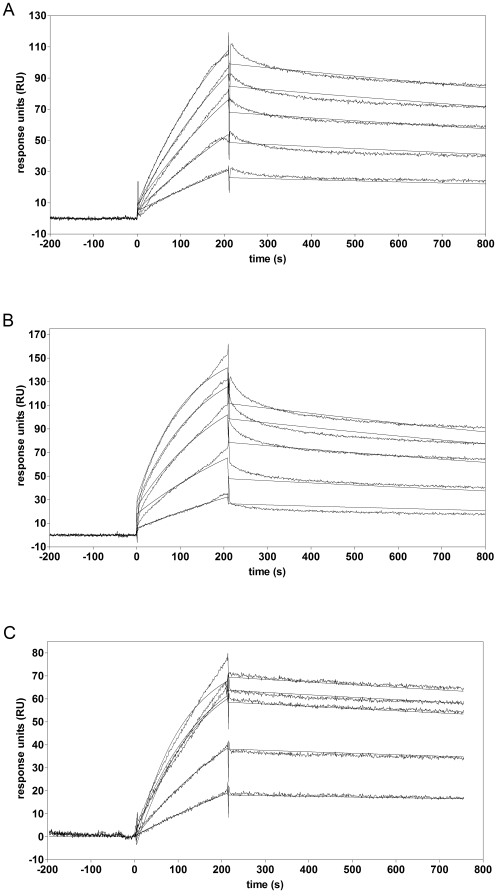
Characterization of the interaction of BMP2, BMP4 and TGFβ1 with Agrin-Nterm by SPR assays. Sensorgrams of the interactions of (A) Agrin-Nterm (660, 1320, 1980, 2640 and 3300 nM) with BMP2; (B) Agrin-Nterm (660, 1320, 2640, 3960 and 5280 nM) with BMP4; (C) Agrin-Nterm (360, 900, 1800, 2160 and 2700 nM) with TGFβ1. Various concentrations of Agrin-Nterm in 20 mM HEPES buffer, pH 7.5, containing 150 mM NaCl, 5 mM EDTA, 0.005% Tween 20 were injected over TGFβ1 or BMPs immobilized on CM5 sensorchips. For each type of experiment, one set of representative data of three parallels is shown. For the sake of clarity the concentrations of Agrin-Nterm are not indicated in the panels; in each case SPR response increased parallel with the increase of Agrin-Nterm concentration.

Surface plasmon resonance analysis revealed that soluble Agrin-Nterm has affinity for immobilized BMP2, BMP4 and TGFβ1 (Figure 4). Evaluation of the sensorgrams has shown that the equilibrium dissociation constants for the interaction of Agrin-Nterm with immobilized TGFβ family members were: TGFβ1, K_D_ = 5,15×10^−8^ M; BMP2, K_D_ = 2,62×10^−7^ M; BMP4, K_D_ = 2,57×10^−7^ M, respectively (Table 1). These relatively high affinities raised the possibility that the Agrin-growth factor interactions may have biological importance. To assess the biological relevance of these interactions we have studied the ability of Agrin-Nterm to block the binding of growth factors to their receptors using SPR in a solution-competition format as well as in luciferase reporter assays.

**Table 1:** Kinetic parameters of the interaction of Agrin-Nterm with different members of the TGFβ family.

Interacting proteins	K_D_ (M)	k_a_ (1/Ms)	k_d_ (1/s)
Agrin-Nterm – TGFβ1*	5,15×10^−8^	3,34×10^3^	1,72×10^−4^
Agrin-Nterm – BMP2*	2,62×10^−7^	1,09×10^3^	2,86×10^−4^
Agrin-Nterm – BMP4*	2,57×10^−7^	1,61×10^3^	4,12×10^−4^

The rate constants of the association and dissociation reactions and the equilibrium dissociation constants of the interactions were determined from surface plasmon resonance measurements with the BIAevaluation software 4.0.

*Proteins marked by an asterisk were immobilized on sensorchips.

### Effect of Agrin-Nterm on binding of BMP2, BMP4 and TGFβ1 to the extracellular domains of their cognate receptors

**Figure 5:**
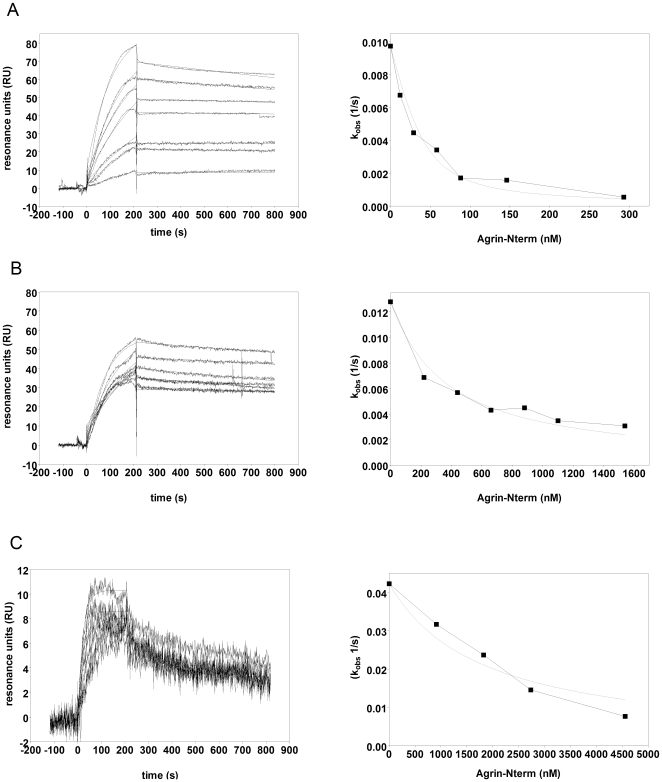
Effect of Agrin-Nterm on the binding of BMP2, BMP4 and TGFβ1 to the extracellular domain of their cognate receptors, monitored by surface plasmon resonance. A) Sensorgrams of the interactions of immobilized ECD of BMPRIA with 40 nm BMP2 preincubated with 0 nM, 12 nM, 29 nM, 58 nM, 88 nM, 146 nM and 293 nM of Agrin-Nterm. B) Sensorgrams of the interactions of immobilized ECD of BMPRIA with 40 nm BMP4 preincubated with 0 nM, 220 nM, 440 nM, 660 nM, 880 nM, 1100 nM and 1540 nM of Agrin-Nterm. C) Sensorgrams of the interactions of immobilized ECD of TGF-βsRII with 4 nm TGFβ1 preincubated with 0 nM, 912 nM, 1824 nM, 2736 nM and 4560 nM of Agrin-Nterm. Growth factors were preincubated with Agrin-Nterm in 20 mM HEPES buffer, pH 7.5 containing 150 mM NaCl, 5 mM EDTA, 0.005% Tween20 for 30 min at room temperature and were injected over CM5 sensorchips containing immobilized ECD of receptors. For the sake of clarity the concentrations of Agrin-Nterm are not indicated in the panels; in each case SPR response decreased parallel with the increase of Agrin-Nterm concentration. Panels on the right indicate the concentration dependence of the inhibitory effect of Agrin-Nterm as monitored by changes in observed association rate, k_obs_.

As shown in Figure 5 preincubation of BMP2 (Figure 5A) or BMP4 (Figure 5B) with increasing concentrations of Agrin-Nterm efficiently decreased the recorded SPR responses and observed association rates, indicating that BMP2-Agrin-Nterm and BMP4-Agrin-Nterm complexes are formed and that these complexes are unable to bind to the ECD of the receptor protein, BMPR1A. Analysis of the data with the method of Nieba et al. [22] has revealed that Agrin-Nterm caused 50% decrease in the observed rate of association (k_obs_) of BMP2 and BMP4 to the ECD of BMPR1A at 12 nM and 345 nM, respectively. In the case of TGFβ1 (Figure 5C) Agrin-Nterm caused 50% decrease in the observed rate of association of TGFβ1 to the ECD of TGF-βsRII at ∼2 µM.

We wish to point out that there is disagreement between the affinities determined in SPR assays in which one of the interacting partners are immobilized (see Table 1) and the affinities determined in solution competition assay formats. Since standard determination of binding constants from on- and off-rates may not reproduce binding constants in solution [22], we assume that the conclusions drawn from the latter experiments have greater biological relevance.

To check whether the interaction of Agrin-Nterm with BMP2, BMP4 and TGFβ1 interferes with the signalling activities of these growth factors we studied the influence of Agrin-Nterm in reporter assays.

### Effect of Agrin-Nterm on growth factor activity of BMP2, BMP4 and TGFβ1

**Figure 6:**
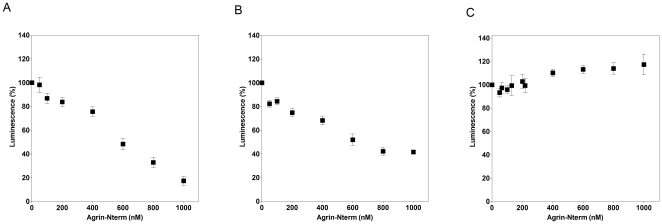
Effect of Agrin-Nterm on growth factor activities. Panel A) HepG2-BRA cells were incubated for 17 hours with 250 pM BMP2 preincubated with different concentrations of Agrin-Nterm. Panel B) HepG2-BRA cells were incubated for 17 hours with 250 pM BMP4 preincubated with different concentrations of Agrin-Nterm. Panel C) Mink lung epithelial cells MLEC-clone32 were incubated for 17 hours with 16 pM TGFβ1 preincubated with different concentrations of Agrin-Nterm; The luciferase activities were normalized to the protein content of the wells and background values obtained from control cells were subtracted. The figure shows the mean values of three parallel experiments.

As shown in Figure 6, Agrin-Nterm efficiently inhibited the activity of BMP2 (Figure 6A) and BMP4 (Figure 6B) in luciferase reporter assays, half maximal inhibition being achieved by ∼0.5 µM and ∼0.6 µM Agrin-Nterm, respectively.

In contrast with BMP2 and BMP4, in the case of TGFβ1 Agrin-Nterm was unable to cause inhibition even at the highest concentrations (1 µM) used; interestingly, Agrin-Nterm caused a slight increase in TGFβ1 activity (Figure 6C).

### Evolutionary history of agrin

We have shown that true orthologs of vertebrate agrins are present in the genomes of Placozoa (*Trichoplax adhaerens*), nematodes (*Caenorhabditis elegans*, *Caenorhabditis briggsae*), some Arthropods (*Apis mellifera*, *Tribolium castaneum*), Echonoderms (*Strongylocentrotus purpuratus*) and Urochordates (*Ciona intestinalis*); see Table S1. Interestingly, no orthologs of agrin could be identified in the completely sequenced genomes of *Drosophila melanogaster* and *Drosophila pseudobscura*.

The fact that agrins are present in *Trichoplax adhaerens* as well as Nematodes and Arthropods indicates that the common ancestor of agrins appeared prior to the divergence of Placozoa and Bilateria. Comparison of the domain architectures of agrins of different groups of Metazoa (see Figure 1) suggests that the common ancestor of these agrins already had an N-terminal laminin binding domain, follistatin-domain(s), EGF domain(s) and laminin G domain(s). Tandem duplication of follistatin-domains, acquisiton of laminin EGF and SEA domains resulted in a domain organization characteristic of vertebrate agrins (see Figures 1 and Figures S1, S2, S3, S4, S5, S6).

Comparison of the sequences of invertebrate and vertebrate agrins have also shown that the sequence motifs essential for the AchR clustering activity of vertebrate agrins are highly conserved in vertebrate agrins, but are not conserved in invertebrate agrins (see Figure 1, Figure S7 and Figure S8), suggesting a major change in the biological function of agrin at the base of vertebrate evolution.

Similarly, the neurotrypsin cleavage sites, highly conserved in vertebrates [23] are not conserved in invertebrate agrins. The region containing the so called α neurotrypsin cleavage site of vertebrate agrins (Figure S9) has no equivalent in invertebrate agrins (see Figure 1). The β neurotrypsin cleavage site of vertebrate agrins is present in the C-terminal region, between the fourth EGF and the third LamininG domain (see Figure 1). Alignment of the eqivalent regions shows high conservation of the β neurotrypsin cleavage site only in vertebrates (Figure S8). It must be pointed out that lack of conservation of neurotrypsin cleavage sites in invertebrates is consistent with the fact that neurotrypsin is restricted to vertebrates [24]. These results thus suggest that the control of agrin's biological function(s) by neurotrypsin evolved only in vertebrates, in parallel with the evolution of agrin's AchR clustering activity.

## Discussion

### The N-terminal part of agrin binds members of TGFβ family

Our observation that agrin binds BMP2, BMP4 and TGFβ1 expands the list of follistatin-domain containing proteins that have affinity for TGFβ family members. Follistatin and the protein encoded by follistatin-related gene (FLRG) bind and inhibit activin and GDF8/myostatin [25]–[28]. These proteins show strong preferential binding for activin over other TGFβ family members, including TGFβ1, BMP4, BMP6, and BMP7 [29], [30]. The follistatin-domains of the multidomain proteins WFIKKN1 and WFIKKN2 appear to be specific for GDF8/myostatin and the closely related GDF11 [17], [31], whereas in the case of agrin the preferred ligands are BMP2, BMP4 and TGFβ1.

Proteins that bind growth factors may control their action in multiple ways: they may act as inhibitors if they prevent their association with cellular receptors, they may serve as a reservoir for growth factors, they may localize their action in the vicinity of the binding proteins. The interplay between these effects is determined by the affinity and concentrations of the various interacting partners. Accordingly, we suggest that binding of growth factors by vertebrate agrins may have multiple functions: agrin may serve as a reservoir of these growth factors, may localize their action and may also inhibit their growth promoting activity.

### Implications of the agrin-growth factor interactions for the development of the neuromuscular junction and CNS synapses

Obviously, the growth factor-binding activity of agrin has relevance for its role in development and maintenance of the neuromuscular junction only if growth factors of the TGFβ family also have a role in the control of synaptogenesis. Although relatively little is known about the role of TGFβs in synaptogenesis in vertebrates, it is noteworthy that in *Xenopus*, Schwann cells were shown to promote synaptogenesis at the neuromuscular junction via TGFβ1 [32] and that in rats BMP2 has been implicated in the modulation of sympathetic neuron growth [33]. In view of our findings it seems plausible to assume that agrin – as a growth factor-binding protein – may influence these processes. Similarly, our findings may be relevant for the observation that the N-terminal half of agrin is involved in agrin's ability to inhibit neurite outgrowth [16], [34]; it seems possible that the N-terminal half of agrin binds and inhibits the growth promoting activity of growth factors thereby inhibiting further neurite outgrowth after synapses have been successfully established.

In the case of some invertebrates, there is clear evidence that signaling by TGFβ family members plays a pivotal role in formation of NMJ [35]. The best-characterized signaling pathway, defined in *Drosophila*, is triggered by Glass bottom boat (Gbb), an Arthropod protein related to BMPs of vertebrates. Gbb acts as a muscle-derived retrograde signal that activates the TGFβ-pathway presynaptically; this pathway includes the type II receptor Wishful thinking, type I receptors Thick veins and Saxophone. Mutations that block this pathway result in small synapses that are morphologically aberrant and severely impaired functionally.

Our results showing that the N-terminal part of agrin binds growth factors may be of particular interest in the context of the recently reported role of this region of agrin for the promotion of dendritic and axonal filopodia, which are considered as precursors of new synapses. Studies with cultured neurons and non-neuronal cells revealed that transmembrane anchored agrin promotes the formation of filopodial protrusions [3], [4], [7], [8] and localized the active region for this effect to the N-terminal part of agrin containing follistatin domains [4], [8]. It is tempting to speculate that the filopodia-promoting effect of the N-terminal part of agrin is mediated by growth factors that are bound in this region.

Our finding that the N-terminal part of agrin binds growth factors may also have important functional consequences for the cleavage of vertebrate agrins by neurotrypsin [23]. Cleavage of vertebrate agrin by neurotrypsin (see Figure 1) separates the C-terminal region (involved in AchR clustering at the NMJ and activity-dependent promotion of dendritic filopodia in the CNS) from the N-terminal moiety (inhibiting neurite outgrowth, promoting axonal and dendritic filopodia and shown here to be involved in growth factor binding). Divorcing the particular functions associated with these regions by activity-dependent proteolytic cleavage may shift the balance between distinct regulatory functions of agrin.

Non-neuronal tissues of vertebrates, such as muscle, heart, kidney also express agrin (isoforms inactive in AchR clustering) but very little is known about the function of agrin in these tissues. We suggest that these agrin isoforms may function as growth factor-binding proteins.

Our finding that the N-terminal part of agrin binds growth factors also has important implications for the biological role of agrin in invertebrates. Despite the ancient origin of agrin practically nothing is known about its function in invertebrates. The fact that the completely sequenced genomes of *Drosophila melanogaster* and *Drosophila pseudoobscura* lack agrin genes (although genomes of other Arthropods, such as *Apis mellifera* and *Tribolium castaneum* do have typical agrins) suggests that insect agrin is dispensable for the synaptogenetic process.

The recent conclusion that *C. elegans* agrin is not involved in synaptogenesis [36] also cautions that functions assigned to vertebrate agrins are not necessarily valid for invertebrate agrins. It is noteworthy in this respect that the sequence motifs of the agrin isoforms that are implicated in AchR-clustering activity are not found in invertebrate agrins although they are highly conserved in vertebrates (see Figure 1, Figure S7 and Figure S8). Similarly, the fact that neurotrypsin cleavage sites are missing from invertebrates (see Figure 1 and Figure S8) suggests that the control of agrin's biological function(s) by neurotrypsin evolved only in vertebrates, in parallel with the evolution of agrin's AchR clustering activity.

The fact that there is an agrin ortholog in the Placozoan *Trichoplax adhaerens*, a simple organism that does not have nerve and muscle cells [37], clearly suggests that ancient agrins had function(s) other than those characterized in vertebrate synaptogenesis. We suggest that agrin's biological function as a growth factor binding protein may be more ancient and more general than its involvement in synaptogenesis. It should be noted that *Trichoplax adhaerens* has multiple members of the TGFβ family and all essential components of the TGFβ signalling pathway are also present in the Trichoplax genome [37].

## Supporting Information

Table S1 Invertebrate agrins. The file contains analyses of sequences from the invertebrate species *Trichoplax adhaerens*, *Caenorhabditis elegans, Apis mellifera*, *Tribolium castaneum*, *Strongylocentrotus purpuratus*, *Ciona intestinalis* that - according to the criteria described in the main text - are orthologs of vertebrate agrins.
(0.28 MB PDF)

Figure S1 Multiple alignment of NtA-domains of agrins. The abbreviations are: agrin_triad_nta - NtA domain of the agrin of *Trichoplax adhaerens*; agrin_caeel_nta - NtA domain of the agrin of *Caenorhabditis elegans*; agrin_strpu_nta - NtA domain of the agrin of *Strongylocentrotus purpuratus*; agrin_cioin_nta - NtA domain of the agrin of *Ciona intestinalis*; agrin_danre_nta - NtA domain of the agrin of *Danio rerio*; agrin_chicken_nta - NtA domain of the agrin of *Gallus gallus*; agrin_mouse_nta - NtA domain of the agrin of *Mus musculus*; agrin_human_nta - NtA domain of the agrin of *Homo sapiens*.
(0.02 MB PDF)

Figure S2 Multiple alignment of Follistatin domains of agrins. The abbreviations are: agrin_triad_fs1, agrin_triad_fs2 - the two follistatin-domains of the agrin of *Trichoplax adhaerens*; agrin_caeel_fs1, agrin_caeel_fs2 - the first two follistatin-domains of *Caenorhabditis elegans*; agrin_apime_fs1, agrin_apime_fs2 - the first two follistatin-domains of the agrin of *Apis mellifera*; agrin_strpu_fs1, agrin_strpu_fs2 - the first two follistatin-domains of the agrin of *Strongylocentrotus purpuratus*; agrin_cioin_fs1, agrin_cioin_fs2 - the first two follistatin-domains of the agrin of *Ciona intestinalis*; agrin_chick_fs1, agrin_chick_fs2 - the first two follistatin-domains of the agrin of *Gallus gallus*; agrin_rat_fs1, agrin_rat_fs2 - the first two follistatin-domains of the agrin of *Rattus norvegicus*.
(0.02 MB PDF)

Figure S3 Multiple alignment of EGF-domains of agrins. The abbreviations are: agrin_triad_egf1, agrin_triad_egf2, agrin_triad_egf3, agrin_triad_egf4 - EGF domains of the agrin of *Trichoplax adhaerens*; agrin_strpu_egf1, agrin_strpu_egf2, agrin_strpu_egf3 - EGF domains of the agrin of *Strongylocentrotus purpuratus*; agrin_cioin_egf1, agrin_cioin_egf2, agrin_cioin_egf3 - EGF domains of the agrin of *Ciona intestinalis*; agrin_rat_egf1, agrin_rat_egf2, agrin_rat_egf3, agrin_rat_egf4 - EGF domains of the agrin of *Rattus norvegicus*.
(0.01 MB PDF)

Figure S4 Multiple alignment of laminin G domains of agrins. The abbreviations are: agrin_triad_lamg1, agrin_triad_lamg2, agrin_triad_lamg3, agrin_triad_lamg4, agrin_triad_lamg5, agrin_triad_lamg6 - Laminin G domains of the agrin of *Trichoplax adhaerens*; agrin_apime_lamg1, agrin_apime_lamg2, agrin_apime_lamg3 - laminin G domains of the agrin of *Apis mellifera*; agrin_strpu_lamg1, agrin_strpu_lamg2, agrin_strpu_lamg3 - laminin G domains of the agrin of *Strongylocentrotus purpuratus*; agrin_human_lamg1, agrin_human_lamg2, agrin_human_lamg3 - laminin G domains of the agrin of *Homo sapiens*.
(0.04 MB PDF)

Figure S5 Multiple alignment of Laminin EGF-domains of agrins. The abbreviations are: agrin_caeel_lamegf1, agrin_caeel_lamegf2 - laminin EGF-domains of the agrin of *Caenorhabditis elegans*; agrin_apime_lamegf1, agrin_apime_lamegf2 - laminin EGF-domains of the agrin of *Apis mellifera*; agrin_strpu_lamegf1, agrin_strpu_lamegf2 - laminin EGF-domains of the agrin of *Strongylocentrotus purpuratus*; agrin_cioin_lamegf1, agrin_cioin_lamegf2 - laminin EGF-domains of the agrin of *Ciona intestinalis*; agrin_chick_lamegf1, agrin_chick_lamegf2 - laminin EGF-domains of the agrin of *Gallus gallus*; agrin_rat_lamegf1, agrin_rat_lamegf2- laminin EGF-domains of the agrin of *Rattus norvegicus*.
(0.01 MB PDF)

Figure S6 Multiple alignment of SEA domains of agrins. The abbreviations are: agrin_cioin_sea - SEA domain of the agrin of *Ciona intestinalis*, agrin_disom_sea - SEA domain of the agrin of *Discopyge ommata*; agrin_danre_sea - agrin of the SEA domain of *Danio rerio*; agrin_chick_sea - SEA domain of the agrin of *Gallus gallus*; agrin_human_sea - SEA domain of the agrin of *Homo sapiens*.
(0.01 MB PDF)

Figure S7 Multiple alignment showing a region of the second LamG domain affected by alternative splicing in vertebrate agrins: the A/y splice site (see Figure 1). The abbreviations are: agrin_triad - agrin of *Trichoplax adhaerens*; agrin_caeel - the agrin of *Caenorhabditis elegans*; agrin_caebr - the agrin of *Caenorhabditis briggsae*; agrin_apime - the agrin of *Apis mellifera*; agrin_trica - the agrin of *Tribolium castaneum*; agrin_strpu - the agrin of *Strongylocentrotus purpuratus*; agrin_cioin - the agrin of *Ciona intestinalis*; agrin_disom - the agrin of *Discopyge ommata*; agrin_chick - the agrin of *Gallus gallus*; agrin_rat - the agrin of *Rattus norvegicus*; agrin_human - the agrin of *Homo sapiens*. Note that vertebrate agrins contain a conserved four-residue insert, KSRK, at the A/y splice site (positions underlined); analysis of genomic sequences revealed that this motif is missing in invertebrate agrins.
(0.02 MB PDF)

Figure S8 Multiple alignment of a region affected by alternative splicing and neurotrypsin cleavage in vertebrate agrins: the B/z splice site and β neurotrypsin cleavage site (see Figure 1). The alignment encompasses the C-terminal part of the fourth EGF domain and the N-terminal part of the third LamG domain. The abbreviations are: agrin_triad - agrin of *Trichoplax adhaerens*; agrin_trica - the agrin of *Tribolium castaneum*; agrin_apime - the agrin of *Apis mellifera*; agrin_cioin - the agrin of *Ciona intestinalis*; agrin_disom - the agrin of *Discopyge ommata*; agrin_chick - the agrin of *Gallus gallus*; agrin_rat - the agrin of *Rattus norvegicus*; agrin_human - the agrin of *Homo sapiens*. Note that vertebrate agrins contain a conserved eight-residue insert, xLxNEIPx, at the B/z splice site (positions underlined); analysis of genomic sequences revealed that this motif is missing in invertebrate agrins. The alignment also includes the β neurotrypsin cleavage site (arrow) of vertebrate agrins (see Figure 1). Note that in vertebrate agrins the β neurotrypsin cleavage site is conserved (positions double-underlined); analysis of genomic sequences revealed that this motif is missing in invertebrate agrins.
(1.09 MB PDF)

Figure S9 Multiple alignment of regions affected by neurotrypsin cleavage (arrow) in vertebrate agrins: the α site (see Figure 1). The abbreviations are: agrin_disom - the agrin of *Discopyge ommata*; agrin_danre - the agrin of *Danio rerio*; agrin_chick - the agrin of *Gallus gallus*; agrin_rat - the agrin of *Rattus norvegicus*; agrin_human - the agrin of *Homo sapiens*. Note that in vertebrate agrins the α neurotrypsin cleavage site is conserved (positions double-underlined); analysis of genomic sequences revealed that this motif is missing in invertebrate agrins.
(0.48 MB PDF)
