# Supplementary material for: Agrin Binds BMP2, BMP4 and TGFβ1
Source: PLoS One. 2010 May 21;5(5):e10758. doi: 10.1371/journal.pone.0010758 (PMC2874008; doi:10.1371/journal.pone.0010758)
Supplement: Table S1 — Invertebrate agrins. The file contains analyses of sequences from the invertebrate species Trichoplax adhaerens, Caenorhabditis elegans, Apis mellifera, Tribolium castaneum, Strongylocentrotus purpuratus, Ciona intestinalis that - according to the criteria described in the main text - are orthologs of vertebrate agrins. (0.28 MB PDF) [file pone.0010758.s001.pdf]

Table S1. Invertebrate agrins. The file contains analyses of sequences from the invertebrate species *Trichoplax adhaerens, Caenorhabditis elegans, Apis mellifera, Tribolium castaneum, Strongylocentrotus purpuratus, Ciona intestinalis* that – according to the criteria described in the main text - are orthologs of vertebrate agrins.

Abbreviations of Pfam domains:
Kazal_1, Kazal_2 = FS; Laminin_EGF = LamEGF; Laminin_G_1, Laminin_G_2 = LamG.

Abbreviations of sequence features:
signal peptide = SP; signal anchor = SA; transmembrane helix = TM

Sequence analysis tools used:

Domain analysis: Pfam A domains were identified by Pfam 23.0 (http://pfam.sanger.ac.uk/)

Signal peptide: signal peptides were identified by Signal P 3.0 (http://www.cbs.dtu.dk/services/SignalP/)

Transmembrane helices: transmembrane helices were identified by TMHMM 2.0 (http://www.cbs.dtu.dk/services/TMHMM/)

Translation initiation sites: Translation initiation sites were predicted by NetStart 1.0 (http://www.cbs.dtu.dk/services/NetStart/)

Quality control of sequences: Errors of sequences were detected and corrected as described in the main text.

Species: ***Trichoplax adherens***
Sequence ID: NCBI Reference Sequence: XP_002113830.1
Sequence name: hypothetical protein TRIADDRAFT_57635
Sequence length: 1556 aa residues

Domains by Pfam:

Pfam A domains	Start	End	E-value
NtA	22	133	0.0066
Kazal_2	169	212	4.6e-07
Kazal_2	242	290	9.2e-08
EGF	297	329	9.5e-06
Laminin_G_2	364	496	4.3e-21
EGF	526	559	8.6e-07
EGF	568	602	1.5e-05
Laminin_G_2	642	770	9.2e-33
EGF	790	821	8.5e-08
Laminin_G_2	862	991	2.3e-32
Laminin_G_2	1048	1173	1.7e-31
Laminin_G_2	1235	1356	1.2e-30
Laminin_G_2	1410	1537	3.8e-34

Region unassigned by Pfam at E-score < 10-2: none

Signal peptide by SignalP 3.0:

	Start	End	Probability
Signal Peptide	1	19	0.950
Signal Anchor	-	-	0.011

Domain Architecture by Pfam with E value =10^{-5} cut-off value:
SP, FS, FS, EGF, LamG, EGF, LamG, EGF, LamG, LamG, LamG, LamG
Domain Architecture by Pfam with E value =10^{-2} cut-off value:
SP, NtA, FS, FS, EGF, LamG, EGF, EGF, LamG, EGF, LamG, LamG, LamG, LamG
Domain Architecture verified by consensus procedure:
SP, NtA, FS, FS, EGF, LamG, EGF, EGF, LamG, EGF, LamG, LamG, LamG, LamG

Alignments of the different domains of *Trichoplax adherens* agrin with the corresponding domains of other agrins are shown in **Figure S1** (NtA domains), **Figure S2** (FS domains), **Figure S3** (EGF domains), **Figure S4** (LamG domains).

Sequence quality by MisPred: No error detected

The domain architecture of the protein is shown in Figure 1.

Deviation of domain architecture from those of secreted vertebrate agrins: The protein contains only two (vs. nine) FS-domains, it lacks laminin EGF and SEA domains. It has three additional Laminin G domains that arose through duplication of the third Laminin G domain.

Species: ***Caenorhabditis elegans***
Sequence ID: GenBank ABF71722.3
Sequence name: Agrin (synaptic protein) homolog protein 1
Sequence length: 1473 aa

Domains by Pfam:

Pfam A domain	Start	End	E-value
Kazal_1	173	220	1.2e-08
Kazal_1	252	301	3.2e-13
Kazal_1	327	371	7.5e-17
Kazal_1	401	445	7e-13
Kazal_1	456	516	9.9e-14
Kazal_1	544	592	7.3e-11
Kazal_2	612	660	2.3e-06
Laminin_EGF	671	720	5.6e-17
Laminin_EGF	723	767	1.7e-07
Kazal_1	802	852	9.7e-09
EB	870	914	0.00069
Laminin_G_2	1109	1238	2.5e-20
Laminin_G_2	1320	1453	6.3e-28

**Region unassigned by Pfam at E-score < 10-2:**	1	172
	915	1108

Signal peptide by SignalP 3.0:

	Start	End	Probability
Signal Peptide	1	22	0.741
Signal Anchor	-	-	0.253

Domain Architecture by Pfam with E value =10^{-5} cut-off value:
SP, FS, FS, FS, FS, FS, FS, FS, LamEGF, LamEGF, FS, LamG, LamG.
Domain Architecture by Pfam with E value =10^{-2} cut-off value:
SP, FS, FS, FS, FS, FS, FS, FS, LamEGF, LamEGF, FS, EB, LamG, LamG.
Domain Architecture verified by consensus procedure:
SP, NtA, FS, FS, FS, FS, FS, FS, FS, LamEGF, LamEGF, FS, EGF, LamG, LamG.

Alignments of selected domains of *Caenorhabditis elegans* agrin with the corresponding domains of other agrins are shown in **Figure S1** (NtA domains), **Figure S2** (FS domains) and **Figure S5** (LamEGF domains).

Sequence quality by MisPred: No error detected.

The domain architecture of the protein is shown in Figure 1.

Deviation of domain architecture from those of secreted vertebrate agrins: The protein contains eight (vs. nine) FS-domains, lacks SEA domain, it has one (vs. four) EGF-domain and two (vs. three) Laminin G domains.

Species: ***Caenorhabditis briggsae***
Sequence ID: NCBI Reference Sequence: XP_002630422.1
Sequence name: CBR-AGR-1 protein
Sequence length: 1469 aa

Domains by Pfam:

Pfam A domain	Start	End	E-value
Kazal_1	154	203	1.4e-06
Kazal_1	233	284	2e-05
Kazal_2	314	354	4.1e-09
Kazal_2	388	428	4.2e-09
Kazal_2	456	499	4e-08
Kazal_2	532	573	4e-06
Kazal_2	598	641	2.5e-05
Kazal_2	675	715	5.2e-08
Laminin_EGF	724	773	4.7e-10
Laminin_EGF	779	840	0.00019
Kazal_2	865	917	1.3e-06
Laminin_G_2	1147	1274	6.6e-16
Laminin_G_2	1317	1450	1.1e-22

**Region unassigned by Pfam at E-score < 10-2:**	1	153
	918	1146

Signal peptide by SignalP 3.0:

	Start	End	Probability
Signal peptide:	-	-	0.005
Signal anchor:	-	-	0.004

Domain Architecture by Pfam with E value =10^{-5} cut-off value:
FS, FS, FS, FS, FS, FS, LamEGF, FS, LamG, LamG.
Domain Architecture by Pfam with E value =10^{-2} cut-off value:
FS, FS, FS, FS, FS, FS, FS, FS, LamEGF, LamEGF, FS, LamG, LamG.
Domain Architecture verified by consensus procedure:
NtA, FS, FS, FS, FS, FS, FS, FS, FS, LamEGF, LamEGF, FS, EGF, LamG, LamG.

Sequence quality by MisPred: the sequence is incomplete at the N-terminal end.

The domain architecture of the protein is similar to that of *C. elegans* agrin (shown in Figure 1) except that it has an additional FS domain.

Deviation of domain architecture from those of secreted vertebrate agrins: The protein lacks SEA domain, it has one (vs. four) EGF-domain and two (vs. three) Laminin G domains.

Species: ***Apis mellifera***
Sequence ID: NCBI Reference Sequence: XP_391941.2
Sequence name: similar to agrin isoform 1
Sequence length: 2397 aa
Database: NCBI

Domains by Pfam:

Pfam A domain	Start	End	E-value
Kazal_2	497	542	1.6e-13
Kazal_1	572	621	4.8e-12
Kazal_1	647	691	2.1e-17
Kazal_1	721	765	1.7e-10
Kazal_1	793	836	3.2e-16
Kazal_1	862	913	1.6e-13
Kazal_1	934	975	1.3e-11
Kazal_2	1044	1089	1.8e-07
Laminin_EGF	1176	1226	3e-24
Laminin_EGF	1229	1259	2.4e-07
Kazal_1	1308	1354	1.2e-14
EB	1437	1477	0.00049
EGF	1493	1525	2.1e-07
Laminin_G_2	1561	1690	1.2e-29
EGF	1810	1828	0.0065
EGF	1835	1867	3.1e-08
Laminin_G_2	1906	2034	5.2e-30
EGF	2056	2089	4.2e-11
Laminin_G_2	2174	2221	3.1e-06

Region unassigned by Pfam at E-score < 10-2:

	Start	End
	1	496
	1691	1809
	2222	2397

Signal peptide by SignalP 3.0:

	Start	End	Probability
Signal Peptide	-	-	0.000
Signal anchor	-	-	0.000

Transmembrane by TMHMM 2.0:
Number of predicted TMHs: 1

	Start	End	Probability
Total prob of N-in:			0.99866
inside	1	436	
TMhelix	437	459	
outside	460	2397	

Domain Architecture by Pfam with E value =10^{-5} cut-off value:
TM, FS, FS, FS, FS, FS, FS, FS, FS, LamEGF, LamEGF, FS, EGF, LamG, EGF, LamG, EGF, LamG

Domain Architecture by Pfam with E value =10^{-2} cut-off value:
TM, FS, FS, FS, FS, FS, FS, FS, FS, LamEGF, LamEGF, FS, EB, EGF, LamG, EGF, EGF, LamG, EGF, LamG.

Domain Architecture verified by consensus procedure:

TM, FS, FS, FS, FS, FS, FS, FS, FS, LamEGF, LamEGF, FS, EGF, LamG, EGF, EGF, LamG, EGF, LamG.

Sequence quality by MisPred:

Violates rule 4: third Laminin G2 domain truncated; C-terminal part mispredicted.

Deviation of domain architecture from those of vertebrate agrins:

The protein lacks SEA domain.

The putative intracellular region of this type II transmembrane protein is much larger than those of vertebrate agrins. Analysis of the 5' region of the predicted agrin gene by NetStart indicates that this is due to misidentification of the translation initiation site in the 5' untranslated region of the gene.

The corrected sequence of *Apis mellifera* agrin protein is:

Name: AGRIN_APIME_CORRECTED, 1901 aa
Sequence:

```
MNGYRKNNFEAYGVYSDPEDDHGFLAEKKTPQYPYKGVVVNVSGESSVIDQKRAKDGRQTEVVANSWCRFTIVLLLLVLLIVIFVFVAGIVLYLNYMSYKPQRPTIEEP
CEKTYCSWGATCVVSENGKPLCQCPTDCPSTSEPVCGSDNVTYTNYCHLRKSSCLERKSTRVKNQGACEIKDPCTKLNCSQGSQCVRSRDGSEASCECLESCPSLGDHE
GSSPVCGTDGTDYPSLCEMNRAACAKGANITMAFQGKCDPCGNVECVEPEICQLDESRQPGCRCGEQCGLEFAPVCGSDGKTYSNECSLRQEACRSRLSLRKVYNGACS
SGINPCDEAKCGPYEQCVINRQGIASCECGAECEPVMRPVCARGGKTYTSLCELKRQACLTRTNIEVAYTGTCGSRGPCSEKICQWGAICAEIGGTAICECPTCPAEFQ
PVCGDDGISYGNECQLRLEGCKHRREIRVLYQGLCNECENKKCEFYAECKTDNGGEAKCVCPSKCEISTKDAAQAAEKICGSDGVTYANECAMKVASCTSQALITVNYV
GDCELCARVECEHGAHCMAGVCVCPDECPESGGEPVCGSDAKTYPSECELQKAACGRDPKLPVLHVIFYGDCGERFAVAALTTMSTPAVVRLATTAVDVTSTQVREACK
NINCDFEATCELGPDDYPRCSCKFDCASISQENMRPVCGSDLRTYSSLCAMKMEACQRQQELRPRPLDLCQGMEVKPCNGDPPLVDADGKEYDCGNGPERRDCPSNSYC
HQTTRLARCCRKAQGTQVVKCSDSWHGCCPDGKTAARGPNGAGCPSLCNCNRLGSVSDTCNPETGQCECKPGVGGLKCDRCMPGYWGLPKISEGHQGCIPCGCSLFGSV
REDCEQMTGRCVCKPDIQGQKCTICTDHNKILTLTGCYSADTIPPIPTSCNELECYSGGQCSEIGGPHCVCPSSCPSDIPSVPVCGSDGQTYDNECELRLYACRHQADV
VTQAFGHCRDDPMVNTDFPVKRYTAVQYTQPAAAISPLSKSTRHLLVPEPDPRYFYTHRAHQETITIDRDNLKHGVAAAYRPTPATIRVVTALLGDLCTDDKDCTIPNS
ECSNGGCICSEGYAETSDRQECFANYVPVTPTEEFRACLSYPCHATSTCIDLPSATFVCICRPNYTGRFCEEMNKRDYEVASFDGKSYVRMNRLKAYHKFSVEVEFKTY
ADNGIILYNQQKSDGTGDFVSLAIVDGHVQFRYNLGNGPVILTSPERVTMKTFHSVAAKRYHKDGVLIFNDGEDVVGQSQGMLKSLDLNQDTFVGNMPTNYSKVYENIG
TNHGFLGCIRKLKINRIHVDLHVGRDKEILETYRVKECGENACANLPCQNGATCQPIFEEDLCNGHECRRIQKRGKGRSKSGMNIVRCKGTHCTLVDRERRSKKNARKR
CATSKCDYDYEVDYENGYEHGNYAVEKYEPPNYRCICPPQFTGRNCEESLDPCIGEPCQHGATCDILPQGGYVCKCPPGRTGEHCEILDAELTELLIPEMSGDGFLELP
CLEGVAKAFSIELWFLTHASDGLLLYNGQLNNGRGDFISLNLVQAKLEFRFNLGSGIANITSPDPVTLDTWHCVRISRLGREGVLQLDDGTVARGLSGSPLTELNLEMP
LYVGGLKHWREIHRLAGARTGLVGAIQRLMVNGKTYQNLAVNVTQHNTEIYDGLPCPSNENPCHNGGVCLPLLNSYLCKCATGYNGLHCEFFMGYDVSTELTERPVRFK
GDNFLQFRHRNGRRRKGQQSNKFELRLRTTHPDGLIAWIGRGKVEHLILSLHGGQVLLTYKSKNEQISLRSRERVDDGVFHQIRASRRRRTSMIQVDDSAPVKVSTEMT
LLTTNGKLFVGGKPGHRGIKGCVSDFVVDKRRLQLGRRRIEYCHDNDV
```

Domains of the corrected bee agrin sequence identified by Pfam:

Pfam A domain	Start	End	E-value
Kazal_2	132	177	1.6e-13
Kazal_1	207	256	4.8e-12
Kazal_1	282	326	2.1e-17
Kazal_1	356	400	1.7e-10
Kazal_1	428	471	3.2e-16
Kazal_1	497	548	1.6e-13
Kazal_1	569	610	1.3e-11
Kazal_2	679	724	1.8e-07
Laminin_EGF	811	861	3e-24
Laminin_EGF	864	894	2.4e-07
Kazal_1	943	989	1.2e-14
EB	1072	1112	0.00049
EGF	1128	1160	2.1e-07
Laminin_G_2	1196	1325	1.2e-29
EGF	1445	1463	0.0065
EGF	1470	1502	3.1e-08

Laminin_G_2	1541	1669	5.2e-30
EGF	1691	1724	4.2e-11
Laminin_G_2	1771	1884	8.3e-25

**Region unassigned by Pfam at E-score < 10-2:**	1	131
	1326	1444

Domain Architecture by Pfam with E value =10^{-5} cut-off value:
TM, FS, FS, FS, FS, FS, FS, FS, FS, LamEGF, LamEGF, FS, EGF, LamG, EGF, LamG, EGF, LamG

Domain Architecture by Pfam with E value =10^{-2} cut-off value:
TM, FS, FS, FS, FS, FS, FS, FS, FS, LamEGF, LamEGF, FS, EB, EGF, LamG, EGF, EGF, LamG, EGF, LamG.

Domain Architecture verified by consensus procedure:
TM, FS, FS, FS, FS, FS, FS, FS, FS, LamEGF, LamEGF, FS, EGF, LamG, EGF, EGF, LamG, EGF, LamG.

The domain architecture of the corrected sequence of bee agrin is shown in Fig.1.

Alignments of selected domains of *Apis mellifera* agrin with the corresponding domains of other agrins are shown in **Figure S2** (FS domains), **Figure S4** (LamG domains) and **Figure S5** (LamEGF domains).

Species: ***Tribolium castaneum***
Sequence ID: NCBI Reference Sequence: XP_001811978.1
Sequence name: similar to agrin
Sequence length: 2027 aa

Domains by Pfam:

Pfam A domain	Start	End	E-value
Kazal_1	237	281	3.7e-15
Kazal_1	311	360	4.4e-16
Kazal_1	386	430	1.6e-18
Kazal_1	460	504	9.6e-15
Kazal_1	532	575	2.8e-17
Kazal_1	601	648	1.2e-15
Kazal_2	668	717	4.7e-07
Kazal_2	803	839	0.00012
Laminin_EGF	928	978	5.5e-20
Laminin_EGF	981	1026	1.3e-05
Kazal_1	1061	1107	6.3e-13
EB	1213	1253	2.6e-05
EGF	1266	1298	4.1e-09
Laminin_G_1	1335	1467	8.6e-29
EGF	1490	1522	7.1e-06
EGF	1529	1561	2e-07
Laminin_G_2	1576	1704	1.6e-29
EGF	1736	1769	1.8e-09
Laminin_G_1	1856	1918	9.6e-13

**Region unassigned by Pfam at E-score < 10-2:**	1	236
	1108	1213
	1919	2027

Signal peptide by SignalP 3.0:

	Start	End	Probability
Signal Peptide	-	-	0.000
Signal anchor	-	-	0.000

Transmembrane by TMHMM 2.0:
Number of predicted TMHs: 1

Total prob of N-in:			0.99981
inside	1	175	
TMhelix	176	198	
outside	199	2027	

Domain Architecture by Pfam with E value =10^{-5} cut-off value:
TM, FS, FS, FS, FS, FS, FS, FS, LamEGF, FS, EGF, LamG, EGF, LamG, EGF, LamG
Domain Architecture by Pfam with E value =10^{-2} cut-off value:
TM, FS, FS, FS, FS, FS, FS, FS, FS, LamEGF, LamEGF, FS, EB, EGF, LamG, EGF, EGF, LamG, EGF, LamG.
Domain Architecture verified by consensus procedure:
TM, FS, FS, FS, FS, FS, FS, FS, FS, LamEGF, LamEGF, FS, EGF, LamG, EGF, EGF, LamG, EGF, LamG.

Sequence quality by MisPred:

Violates rule 4: third Laminin G2 domain truncated; C-terminal part mispredicted.

Deviation from related agrins:

The protein lacks SEA domain.

The putative intracellular region of this type II transmembrane protein is larger than those of related vertebrate agrins. Analysis of the 5' region of the predicted agrin gene by NetStart indicates that this is due to misidentification of the translation initiation site in the 5' untranslated region of the agrin gene.

The corrected sequence of *Tribolium castaneum* agrin protein is:

Name: AGRIN_TRICA_CORRECTED, 1885 aa
Sequence:

```
MRYTNGPQSAASMVASYGVYNDATLRADEDEDGFIPRANVYPGVVVNAAGDSSVLRRGQEAAPAESYSNSCLISWCRPTILLLILVLLVVVFVLVSGILLYFNYLMHKP
RQHYIEEPCEKTFCAWGAQCITGPDGRAMCQCPTHCKQKVDLVCGTDGKTYANRCQLRVASCKARLNTRVKHDGECEQNDPCRDKQCGFGARCVVSPDGRNASCVCPDK
CPSYGDHTTSRPVCGSDGIDYRNQCELQKAACTSNTNITIKFLGKCDPCAGVECAEPEVCQLDEHRNPVCRCGDSCPLEFTPVCGSDGKTYSNECTLRQEACRARKNLH
IIYRGKCSSGINPCMSVRCTLGEECAINKFGIARCQCPPSCEPIMRPVCSKDGRTFPSECELKRTACLTRTTIEISYSGVCGEKGPCSEYQCQNGATCVERFGVAHCEC
PVCPAEFEPVCGSDGISYGNECKLRLEACKHPRNITVLYDGPCNGCETKKCDFYSVCERDSATEGRCVCPKSCTDPELNDGTVCGTDGVTYANECELRMTSCKMKQFIL
VAYKGNCDLCQGVECKYGGRCEAGKCVCPTNCEGSGDEPVCASNMMTFPNECELQKAMCLQPTNSPPLSVVFYGDCREKFPVAGSLSTTLSPFTAQTNNIGPGTDLNTV
GPENTIPDLSTVEKEACRDIHCDFEATCELGPDNFPRCTCQFDCASAALVSASKPVCASDLQIYPSLCAMKMEACQRQEELRLRPLDLCQGMEVKPCNGEKPLTDPLTG
KELDCGSGPKRQDCPSGSYCHQTTRFSRCCPKDQGLLEQKSCEDSWHGCCPDGKTAAQGPNHAGCPSLCGCNKLGSYSDTCDPETQQCRCRPGVGGAKCDRCEPGYWGL
PKISSGYHGCIPCGCSTFGSVRDDCEQMTGRCVCNPGVQGQKCTVCTSHDKVLGPNGCVPADLTTSPPTTCKELTCYFGATCVERGGFAICECHTECPQENDAQVVCGS
DGQTYASACELRQVACRTQKDIVVQAFGTCKDDMFPSTDWPIRRYTPLQFTQPDDSNSPLSKSTRHLLVPDPRYYYGSSGSIMRPVQGVSYPAETDNKAAFEINVSATG
PNAIYNTRGGNYAPAYRPTPATVRVITALLGDLCSDNSDCMIMYSHCVSGACTCLPNHSESSDRQECIADPNPTDEYRACSSSPCHHGSTCVDLPAATFTCVCETNFTG
SLCETEVIHKQYNTPAFHGRSYVKLKPLKAYHKLSIEVEFKTHSHDGLLLYNQQKPDGLGDFVSLAIVNGFVEFKYNLGNGHVLIRSVDKIQLGVFHRVVIKRYHRDGI
LKLDEGEDVAGQAKGTLKALDLLEDTYVGFLPSNYTRVYENIGTAKGFRGCIRKLKIGRRPIELHVNRDDWVLGAEAVSECGDSPCSAAPCANDAKCALITPETYRCEC
GPHYRGEFCEKPIEPCRSNPCKFGSTCTPLSPDAYICECPPGRTGTRCEMEDKYMGIVNPEFSGSSYIRLPRLEGVRKTFSIEVYFMPKAANGLILYNGQLKNGRGDFI
SLNLARGHLQFRFNLGSGIANLTTKETVNIGKWHWARIFRDGREGILQLDNSSIVRGYSGTPLTELNLELPFYIGSLSEWDEVHRLSGASKGYKGVIQRILLNGKPLPI
SAKLPDCSFDSNRNGCAFNVGSYDGLPCPISKNPCLNNGLCVPELDQFICKCPSNTKGKYCEISQEETPAIKFNGATFLQFRNRGYRRRKPERGNRYEIKLRTFASDAL
LLWRSKSRSIREDYFSIAVVNGYPEISYNLGKQDRMFFRSKTKIDDGKWHTIQVRRRKRVGFISIDGEPPIKGLSKYGAISLRTNSKLWIGGTANLPQGLPSAYYKGFE
GCIQHILVNAKPLDMVSNNELNKVNFCHDNEI
```

Domains of the corrected sequence of beetle agrin identified by Pfam:

Pfam A domain	Start	End	E-value
Kazal_1	141	185	3.7e-15
Kazal_1	215	264	4.4e-16
Kazal_1	290	334	1.6e-18
Kazal_1	364	408	9.6e-15
Kazal_1	436	479	2.8e-17
Kazal_1	505	552	1.2e-15
Kazal_2	572	621	4.7e-07
Kazal_2	707	743	0.00012
Laminin_EGF	832	882	5.5e-20
Laminin_EGF	885	930	1.3e-05
Kazal_1	965	1011	6.3e-13
EB	1117	1157	2.6e-05
EGF	1170	1202	4.1e-09
Laminin_G_1	1239	1371	8.6e-29
EGF	1394	1426	7.1e-06
EGF	1433	1465	2e-07
Laminin_G_2	1503	1631	1.6e-29
EGF	1663	1696	1.8e-09
Laminin_G_1	1736	1867	6e-30

Region unassigned by Pfam at E-score < 10-2:	Start	End
	1	140
	1108	1213

Domain Architecture by Pfam with E value =10^{-5} cut-off value:

TM, FS, FS, FS, FS, FS, FS, FS, LamEGF, FS, EGF, LamG, EGF, LamG, EGF, LamG

Domain Architecture by Pfam with E value =10^{-2} cut-off value:

TM, FS, FS, FS, FS, FS, FS, FS, FS, LamEGF, LamEGF, FS, EB, EGF, LamG, EGF, EGF, LamG, EGF, LamG.

Domain Architecture verified by consensus procedure:

TM, FS, FS, FS, FS, FS, FS, FS, FS, LamEGF, LamEGF, FS, EGF, LamG, EGF, EGF, LamG, EGF, LamG.

Species: ***Strongylocentrotus purpuratus***
Sequence ID: NCBI Reference Sequence: XP_001194095.1
Sequence ID: NCBI Reference Sequence: XP_791472.2
Sequence name: similar to agrin
Sequence length: 1397 aa
and
Sequence ID: NCBI Reference Sequence: XP_791521.1
Sequence name: similar to AGRN protein
Seequence lengthL 306 aa

Domains of XP_791472.2 by Pfam:

Pfam A domain	Start	End	E-value
Kazal_1	54	98	4.8e-16
Kazal_1	126	170	2.5e-16
Kazal_1	198	242	2.1e-19
Kazal_1	267	303	6e-11
Kazal_2	342	386	7.8e-12
Kazal_1	394	442	1.7e-12
Kazal_1	467	511	2.3e-16
Kazal_2	534	582	2.2e-11
EGF	611	635	0.0033
Kazal_1	634	678	3e-17
Laminin_EGF	766	816	2e-18
Laminin_EGF	819	863	2.6e-09
Kazal_1	902	949	4.5e-10
Laminin_G_1	1079	1220	2e-25
EGF	1242	1276	7.7e-06
EGF	1284	1316	0.00025
Laminin_G_1	1351	1391	5.5e-15

**Region unassigned by Pfam at E-score < 10-2:**	950	1078

Signal peptide by SignalP 3.0:

	Start	End	Probability
Signal Peptide	1	19	0.999
Signal Anchor	-	-	0.000

Sequence quality by MisPred:

XP_791472.2 violates rule 4: second (terminal) LamG domain truncated; C-terminal part mispredicted.

Deviation from vertebrate agrins:

1. The predicted protein has ten (vs. nine) follistatin domains has two (vs. four) EGF domains and lacks SEA domain.
2. The predicted protein has two (vs. three) LamG domains.
3. Unlike other secreted agrins, XP_791472.2 lacks an NtA domain.

Domains of XP_791521.1 by Pfam:

Pfam A domain	Start	End	E-value
Laminin_G_2	2	65	2.1e-07
Laminin_G_2	163	286	7.9e-30

Signal peptide by SignalP 3.0:
Prediction: Non-secretory protein
Signal peptide probability: 0.000
Signal anchor probability: 0.000

Transmembrane by TMHMM 2.0:
Number of predicted TMHs: 0

Total prob of N-in:			0.01187
outside	1	306	

Sequence quality XP_791521.1 by MisPred:

Violates rule 1 (has extracellular domains but lacks signal peptide/anchor or transmembrane sequences). Violates rule 4: first LamG domain truncated; N-terminal part mispredicted.

Correction of the sequence of sea urchin agrin:

1. The mispredicted C-terminal part was corrected; the N-terminal and C-terminal parts of the second LamG domain of the corrected agrin sequence correspond to regions of the XP_791472.2 and XP_791521.1, respectively. The third LamG domain of the corrected sequence, missing from XP_791472.2, is found on XP_791521.1.

2. The NtA domain of the corrected sequence of sea urchin agrin was identified by searching the genome of *S. purpuratus* with the sequences of NtA domains of other chordates. The search has identified the exon encoding a signal peptide and the NtA domain of sea urchin agrin on the genomic contig of *S. purpuratus*, NW_001292972.1.

The corrected sequence of *Strongylocentrotus purpuratus* agrin is:

Name: AGRIN_STRPU_CORRECTED, 1897 aa
Sequence:

```
MQLLAFFTSVLVAVACLLAMEQLPGVTGVQIRRNGKTCQEYPVEKREEIANVVISGYIHRIMRKPRTIMYDCDIEVVRVFKGSEALYSNVVPPMDGNTIQIGGFGDPAI
CDNAVTPGETRIFMMNKNGNGHLMLNSSVIRITLNNLVQAEAAVFGPCDMVLCIFGAVCEENEQGRPQCICDRQCPDMMAPVCGSDGTTYLSECFLDKASCEQKKRVYV
ASQGSCDEQDPCEGVQCSFGSECMIEGDRATCECPNACPLIYSPICGSDGVSYGNTCEMEAASCRQQKEITLVNEGMCVEVDPCDNVTCNFGASCVVEGAVASCLCPEI
CLESYNPVCGSDGVDYNNECDLNAAACSQQKSVTVVFQGLCDPCQEVEYENSVCKLDRNREPQPGCDSNCPNSVINPVCGSDGVTYDNDCEINRAACLSNLEDILITFT
EGPCEDGTHPCEEFTCDYGQCLVDDAGMPQCVCTPCPEVFTPVCGSDGLTHSSMCHMEEASCMERTDITLAKEGVCDGSNIIGCQTPACNKTEGAVSPVCGTDGNNYPG
LCALQEAACEAGIDIQVAINGPCESCETTNCSHGSFCQMTPDGPTCTCSDHCQTINLPVCGSDGETYASECKLNVMACNARKNITVVSYGACEDCVGVTCETERFNQVC
YQGMCVCQESCPMSRSDEDMVCGSDQVTYDTVCHLKMSACQAESNLTVEYYGPCDEFSGSGTEFPDYSGSGATPPNEFEFDLCDETSCSFGGICRPLSADTYECICKFN
CPAVRLPVCGSDGATYGNECQLKEAACEQQSSIVLEKIGTCEDVEMEPCDGESPLVNEATMEAYTCQEDNGGEDCPSGSYCHIHPLGRFSACCAEQTSQPSCMESVFGC
CPDNQTEALGSNGEGCPSVCNCNPLGSYSVYCEPSNLQCPCKPGVGGKQCDRCEPGYYDFLSLERNGIGCKTCGCSSYGSRRDDCDQTSGACRCRRKAIGLKCNMCPEG
LTMTPMGCMTEEEQMMTSAITCSNLTCPFSAQCQESTVEGQNATCVCPTSSSCNTDVIQVVCGDNGETYPSRCQLQVFACKEQRNIMVQNEGACETVIVTPNEIRPGCE
ESQFRCCPDQVTYATGPNNEGCSDASGTPPLAADQNAGQASASSPAPPSAATVALTTDAVVPSTKVSSTTLSVAPFVSTALPTDSVLPTTTIVTIVLGCDSSPCQHGGT
CQNDEIAPGFRCICPLGKGGPVCNEVVTFTTPSFAGDSYLAYPEMDAFMEVEIVIEFQPGATEGVLLYEAQTAEGNGDFISLAIVNNQVEFRFLATDQSNGFDLGSAEP
VVITSTVDLQLMTWHRLRAYRSRREGSLSVDGEPEVTGTSEGVSGALNLGEDLFIGYAVPPEVGLRLANTNQGFVGCIRYVEINSQELDISSSGSSVEYGANVGECGND
PCQSKEMPCFNNGLCEALNAESYRCICQGDFFGTLCGDVLVDQCEGHMCHEESTCVALPEGGYRCDCPDGRMGDMCTEEINKVVVPGFAGNSYMQLPSLMMPDDSVIDV
EFLTSSPDGVIFYNGQTADGRGDFISLNMRDGYLEFRYDLGSSIAEIKSVDRLALNEWHAVRVIRMGKSGEMILNDLPPVKGTSPPGASQLNLRQPLFIGGVRSYGEVS
RRAAITDGLNGAVRRFVVNEVDYSTLKDFAEAKVNVEEFREDTMNMIPTAPRKLPEMPVTTPRPPVVATRARPAMTTASVVVINTDDPLRFDGQTSIQYYNGVSKKQRA
LRTHQIQLSFKTAEPNGALFWNGVGNADFQAVGVSDGYVEYAYNLGRGITRIRTTQKVDNNKWHTVIITRNLIDASLQVDNEEPVMGQSRAGASQLDTDGFLYLGQGVN
VPGSMESSYTYYTGCIQDVLLDEVPLHLYENAQGEKPSLFCSEP
```

Domains of the corrected sequence of sea urchin agrin identified by Pfam:

Pfam A domains	Start	End	E-value
NtA	37	157	6.8e-22
Kazal_1	180	224	4.8e-16
Kazal_1	252	296	2.5e-16

Kazal_1	324	368	2.1e-19
Kazal_1	393	429	6e-11
Kazal_2	468	512	7.8e-12
Kazal_1	520	568	1.7e-12
Kazal_1	593	637	2.3e-16
Kazal_2	660	708	2.2e-11
Kazal_1	760	804	3e-17
Laminin_EGF	892	942	2e-18
Laminin_EGF	945	989	2.6e-09
Kazal_1	1028	1075	4.5e-10
EGF	1189	1222	1.6e-08
Laminin_G_1	1256	1397	2e-25
EGF	1419	1453	7.7e-06
EGF	1461	1493	0.00025
Laminin_G_2	1528	1656	1e-32
Laminin_G_2	1754	1877	7.9e-30

Region unassigned by Pfam: 1076 1188

Domain Architecture of the corrected sequence of ***Strongylocentrotus purpuratus*****:**
SP, NtA, FS, FS, FS, FS, FS, FS, FS, FS, FS, LamEGF, LamEGF, FS, EGF, LamG, EGF, EGF, LamG, LamG.

The domain architecture of the corrected sequence of sea urchin agrin is shown in Fig.1.

Deviation from vertebrate agrins:

The protein has ten (vs. nine) follistatin domains has three (vs. four) EGF domains and lacks SEA domain.

Alignments of selected domains of *Strongylocentrotus purpuratus* agrin with the corresponding domains of other agrins are shown in **Figure S1** (NtA domain), **Figure S2** (FS domains), **Figure S3** (EGF domains) **Figure S4** (LamG domains) and **Figure S5** (LamEGF domains).

Species: ***Ciona intestinalis***
Sequence ID: NCBI Reference Sequence: XP_002123658.1
Sequence name: similar to transmembrane agrin
Sequence length: 2114 aa

Domains by Pfam:

Pfam A domains	Start	End	E-value
NtA	61	187	1.2e-06
Kazal_1	227	273	7.2e-13
Kazal_2	307	352	1.9e-07
Kazal_1	384	430	3.7e-12
Kazal_2	678	722	6.4e-08
Kazal_1	748	792	6.5e-19
Kazal_1	829	873	6.3e-14
Laminin_EGF	904	955	1.3e-20
Laminin_EGF	958	988	4.1e-08
Kazal_1	1038	1085	9.9e-15
SEA	1292	1401	0.0017
Laminin_G_1	1455	1588	2.7e-34
EGF_2	1631	1645	0.019
EGF	1662	1695	5.8e-11
Laminin_G_1	1737	1868	3.8e-32
EGF	1888	1919	9.8e-08
Laminin_G_2	1966	2094	9.7e-29

**Region unassigned by Pfam at E-score < 10-2:**	431	677
	1086	1291

Signal peptide by SignalP 3.0:
Prediction: Signal anchor
Signal peptide probability: 0.422
Signal anchor probability: 0.462
Max cleavage site probability: 0.224 between pos. 43 and 44

Transmembrane by TMHMM 2.0:
Number of predicted TMHs: 1

Total prob of N-in:			0.98326
inside	1	20	
TMhelix	21	43	
outside	44	2114	

Domain Architecture by Pfam with E value =10^{-5} cut-off value:
TM, NtA, FS, FS, FS, FS, FS, FS, LamEGF, LamEGF, FS, LamG, EGF, LamG, EGF, LamG
Domain Architecture by Pfam with E value =10^{-2} cut-off value:
TM, NtA, FS, FS, FS, FS, FS, FS, LamEGF, LamEGF, FS, SEA, LamG, EGF, EGF, LamG, EGF, LamG.
Domain Architecture verified by consensus procedure:

TM, NtA, FS, FS, FS, FS, FS, FS, LamEGF, LamEGF, FS, SEA, LamG, EGF, EGF, LamG, EGF, LamG.

Sequence quality by MisPred: no error detected

Deviation from vertebrate agrins:

The protein contains only seven (vs. nine) FS-domains and only three (vs. four) EGF domains.

The protein is predicted to be a type II transmembrane protein but unlike transmembrane forms of other agrins it has an NtA domain. Analysis of the 5' region of the predicted agrin gene by NetStart indicates that this discrepancy is due to the fact that the translation initiation site was mispredicted in the 5' untranslated region of the gene. The highest probability translation initiation site corresponds to Met-22 of XP_002123658.1

The corrected sequence of *Ciona intestinalis* agrin corresponds to a secreted protein:

Signal peptide by SignalP 3.0:

	Start	End	Probability
Signal Peptide	1	22	0.998
Signal Anchor	-	-	0.000

Domain Architecture of the corrected sequence of *Ciona intestinalis* agrin**:**

SP, NtA, FS, FS, FS, FS, FS, FS, LamEGF, LamEGF, FS, SEA, LamG, EGF, EGF, LamG, EGF, LamG.

The domain architecture of the protein is shown in Fig.1.

Alignments of selected domains of *Ciona intestinalis* agrin with the corresponding domains of other agrins are shown in **Figure S1** (NtA domains), **Figure S2** (FS domains), **Figure S3** (EGF domains), Figure S**5** (LamEGF domains) and **Figure S6** (SEA domains).

The corrected sequence of *Ciona intestinalis* agrin is:

Name: AGRIN_CIOIN_CORRECTED, 2093 aa
Sequence:

```
MYIRTLILLLSFLYLINTGAECYRIKRAVMLSMDPQPAVECLDRTTTEKNSTSTVILTGTVQYCDVIRAGSYKCNIKIWRVMKGNSLAMNLITIQKQGILTSMYKKKYV
DVYGLGNSAFCKSNVERADTRIFFLNKLNNRLVVSASLDRITLKNLENTQKVIEGKRIKEEPKKPRDPCQEVLCGYGASCQAVDEDTTTCVCPPVSCSASPTTEDILAP
ICGSDGVTYPNECRLRAAECMAQHRIKIKATTSCEPVDDRLCSTVTCDFNAACVVRPSDDGSLAPECECPTCENVAVEPVCGSDQRSHRSSCDLRRASCVEGRVITVVR
NSACNPCDIFNIESGIFLPMCSGSCTLDGNGEPQCCETLKCAANETELVCGSDGNTYENECSLRRSACKTAKSITVIKQGSCPTKKEMTAVRGSLSIQADENCPGNCDL
QNKEFTDFLTRMDTSVNQNLMSQNNQDIDGKTLGDFQITEVRQGSAILLFRAYSSTTSQPIAVNSNANSNNEWIYDRLYSDDNGALKVVARENGLNLVTARNPRDDFVV
EDVCMDLQCEHGAMCKITKSGSTLTPSCSCENVGAAESLPVTHACKQATDGNGVHRIFMNEAEARGESCTSNQILDFKDICQEAPIDPCAEIGGCSGVGEACIVMNGVG
SCQCITCGEEYQPVCTNDGQTFRSHCEVRRHNCLSKTNLIILSIGSCESQCDDIKCWYGAQCEVIAQGTTCVCPTVCVKTYLPVCGSDGQTYSNECEMVVAACPQKLEV
TVAHAGPCDEPTHEGSGSGAGDECGCLFGASCDTTIDDEDANCVCNFNCEAIGVAVCGSDGKTYPNMCELEKAQCNQQTPISLVSKGICKVGCVQSRYGCCEDGTTPAK
GVARSGCPEKCLCNEHGSYGNACNPTTGQCVCRPGVGGLRCDRCRPGYWNFRALAEKLFTGCMSCRCHEFGSTRDDCDQMTGRCSCKVGVTGLKCTDCVSDGSWVTSSV
CDNAATAAPSAVSCEDLVCRVGQECVEINNAFECQCPSLTSCDNMDEAVVCGTNGITYADRCQLKVLACKVGVNVTVAHEGACMSNLEPLVEGPIVRPPLVPVTIETTR
MVQITTTTHPVPVVEPEPTPNAKPEPEPTSKPEPEPEPTPNAKPEPTPKSEPEPEPTSKPEPEPEPTSNPEPEPTPNAKPEPTSNPEPEPERTTKTPLVPKSEPETLAT
SKATPATTVLPVPTTTLSVTKTPKTTAATDEVECMYGDDEDCGGSGSGEGEVDNTEAPSVIPEIPEVPTPAAVQFIGQGTFYSIDGNSLRHSKSLDVPGSEEFINYSNL
VEAEIMSLISNPPLLDSVRAVRISSFRSASIFWGGVIVTFELHLTSGSDAGAIQDALNAASSENRYFTISELTDAERMLEEYTTPVPALSNYTEDGPWTVPHFSGASYA
EFRKVNAFSEITIQLKFRSADPEGILFYSGQLNNGRDFISLAINNGYVEFRFDMGSGMLKLRSKRPINSTQWHTIVARRIRRDGMLQVDADPPVQGTSPGHASGLNLDS
NFFVGGFNTYLEDQRYQKQTGVDKGLTGCIEEVRINENQLNITSNSDHCVSTFRLAECGASPCIPNPCQHSANCFITMQANIFTNKCECKDNYEGETCQRENTGLSVKK
KPNNPCSPNPCQGGAKCIEMPGEEEFTCKCPPGRSGSLCMTNQSAALQGPSFMPAFAGDSYLELPSLGKDVRSIMSIEILFYSNQPDGLIFYNGQKKSGKGDFVSLNLK
NGFLEFKYNLGQGAANIRSANPVSLNEWHIVVLSRAMRTGDLSLDNFDPVYGTSPSQHSFLDLKQPMYVGGFPDGVKFNPEAGVTTGLSGALQKFQVNGVNLPISPAGS
VSYFNVIAFNAHTCYRNPCDNGGVCHPRGAEYMCVCLPYYTGDNCEQEHSTDLLQDEQATAIYLDGTTKIMYRNAVKAISRARTHNNYEIVFRTTARHGLLLMVGKARE
GVDYIALAIHDGRLHLRFDLGSGPAHVISDQQINNGEWTTVKVNRKMNIGSLQVNNGLIKTATSPGLTNHLNSDGMLWLGGVDYRPRGIGLYKSFFVPFVGCVASAKIH
DESIDLRADAFNSPVVRSCNGR
```
