# Supplementary material for: Agrin Binds BMP2, BMP4 and TGFβ1
Source: PLoS One. 2010 May 21;5(5):e10758. doi: 10.1371/journal.pone.0010758 (PMC2874008; doi:10.1371/journal.pone.0010758)
Supplement: Figure S1 — Multiple alignment of NtA-domains of agrins. The abbreviations are: agrin_triad_nta - NtA domain of the agrin of Trichoplax adhaerens; agrin_caeel_nta - NtA domain of the agrin of Caenorhabditis elegans; agrin_strpu_nta - NtA domain of the agrin of Strongylocentrotus purpuratus; agrin_cioin_nta - NtA domain of the agrin of Ciona intestinalis; agrin_danre_nta - NtA domain of the agrin of Danio rerio; agrin_chicken_nta - NtA domain of the agrin of Gallus gallus; agrin_mouse_nta - NtA domain of the agrin of Mus musculus; agrin_human_nta - NtA domain of the agrin of Homo sapiens. (0.02 MB PDF) [file pone.0010758.s002.pdf]

```
agrin_triad_nta    CSPSPLQQRI.....SNADYVITAKVLN...INRNQQQPTYSSNVEV
agrin_caeel_nta    CLKPRPTRSYIHSLFENSDVIIAGVITGISY....RVDSLYIENITV
agrin_strpu_nta    CQEYPVEKRE.....EIANVVISGYIH..RIMR.KPRTIMYDCDIEV
agrin_cioin_nta    CLDRTTTEKN.....STSTVILTGTVQYCDVIRAG....SYKCNIKI
agrin_danre_nta    CSEKELEKRE.....EEANVVLTGTVE..EILNMDPVHNTYSCKVRV
agrin_chicken_nta  CPERELQRRE.....EEANVVLTGTVE..EIMNVDPVHHTYSCKVRV
agrin_mouse_nta    CPERALERRE.....EEANVVLTGTVE..EILNVDPVQHTYSCKVRV
agrin_human_nta    CPERALERRE.....EEANVVLTGTVE..EILNVDPVQHTYSCKVRV

agrin_triad_nta    I..EVFKPPRLSLPL................SITIDNFGNTRICRSN
agrin_caeel_nta    IPRRIYKGREYV...............EENKNIVIGNILETEPCAHR
agrin_strpu_nta    V..RVFKGSEAL.......YSNVVPPMD.GNTIQIGGFGDPAICDNA
agrin_cioin_nta    W..RVMKGNSLAMNLITIQKQGILTSMYKKKYVDVYGLGNSAFCKSN
agrin_danre_nta    W..RYLKGKTMV.......NGEVL..LDGGNKVMIGGFGDPHICDNQ
agrin_chicken_nta  W..RYLKGKDIV.......THEIL..LDGGNKVVIGGFGDPLICDNQ
agrin_mouse_nta    W..RYLKGKDVV.......AQESL..LDGGNKVVIGGFGDPLICDNQ
agrin_human_nta    W..RYLKGKDLV.......ARESL..LDGGNKVVISGFGDPLICDNQ

agrin_triad_nta    VNTNDTRILLFN........GSSQKYVLSTSFEYVTEERLKLIRTA
agrin_caeel_nta    LLASDVRIFSLT........NQQGVFLLDAPMIRLTGNEEERNKVA
agrin_strpu_nta    VTPGETRIFMMN.......KNGNGHLMLNSSVIRITLNNLVQAEAA
agrin_cioin_nta    VERADTRIFFLNKL........NNRLVVSASLDRITLKNLENTQKV
agrin_danre_nta    VATGDTRIFFVNLAPEYMWPNHKNELMLNSSLMRITLRNLEEVEHC
agrin_chicken_nta  VSTGDTRIFFVNPAPQYMWPAHRNELMLNSSLMRITLRNLEEVEHC
agrin_mouse_nta    VSTGDTRIFFVNPAPPYLWPAHKNELMLNSSLMRITLRNLEEVEFC
agrin_human_nta    VSTGDTRIFFVNPAPPYLWPAHKNELMLNSSLMRITLRNLEEVEFC
```
