# Supplementary material for: Agrin Binds BMP2, BMP4 and TGFβ1
Source: PLoS One. 2010 May 21;5(5):e10758. doi: 10.1371/journal.pone.0010758 (PMC2874008; doi:10.1371/journal.pone.0010758)
Supplement: Figure S2 — Multiple alignment of Follistatin domains of agrins. The abbreviations are: agrin_triad_fs1, agrin_triad_fs2 - the two follistatin-domains of the agrin of Trichoplax adhaerens; agrin_caeel_fs1, agrin_caeel_fs2 - the first two follistatin-domains of Caenorhabditis elegans; agrin_apime_fs1, agrin_apime_fs2 - the first two follistatin-domains of the agrin of Apis mellifera; agrin_strpu_fs1, agrin_strpu_fs2 - the first two follistatin-domains of the agrin of Strongylocentrotus purpuratus; agrin_cioin_fs1, agrin_cioin_fs2 - the first two follistatin-domains of the agrin of Ciona intestinalis; agrin_chick_fs1, agrin_chick_fs2 - the first two follistatin-domains of the agrin of Gallus gallus; agrin_rat_fs1, agrin_rat_fs2 - the first two follistatin-domains of the agrin of Rattus norvegicus. (0.02 MB PDF) [file pone.0010758.s003.pdf]

```
agrin_triad_fs1 CSEIVCRMGATCSNEIQEG.....QCSC..IDCHNLTKSE....P
agrin_triad_fs2 CNNITCENYSICRLESNGITDNNPTCRCPTNVC......SLYYNP
agrin_caeel_fs1 CEQSLCPFGSKC.......GLKTGVCEC.KARCRVVTDV......
agrin_caeel_fs2 CEDLRCGPGEDCVVNQINGILLA.KCVCP.TQCPNYGDSVE.SSP
agrin_apime_fs1 CEKTYCSWGATC..VVSENGK.P.LCQCP.TDCPS......TSEP
agrin_apime_fs2 CTKLNCSQGSQCVRSR.DG.SEA.SCECL.ESCPSLGDHEG.SSP
agrin_strpu_fs1 CDMVLCIFGAVCEEN..EQGR.P.QCICDRQ.CPD......MMAP
agrin_strpu_fs2 CEGVQCSFGSEC..MI.EGDR.A.TCECP.NACPL......IYSP
agrin_cioin_fs1 CQEVLCGYGASCQAV..DEDT.T.TCVCPPVSCSASPTTEDILAP
agrin_cioin_fs2 CSTVTCDFNAACVVRPSDDGSLAPECECP..TCENVA.....VEP
agrin_chick_fs1 CRGMLCGFGAVCERSPTDPSQ.A.SCVCKKTACPV......VVAP
agrin_chick_fs2 CAEVTCSFGSTCVRSA.DG.QTA.GCVCP.ASCSGVAE.....SI
agrin_rat_fs1   CRGMLCGFGAVCEPSVEDPGR.A.SCVCKKNACPA......TVAP
agrin_rat_fs2   CANVTCSFGSTCVPSA.DG.QTA.SCLCP.TTCFGAPD.....GT

agrin_triad_fs1 VCASDLKVYSNTCQLERQSCLTGT...QLNILPRDNC
agrin_triad_fs2 ICGNNNKTYYTQCHMIQDSC...QNNITVIKSYGGEC
agrin_caeel_fs1 VCGSDHVSYSSFCHLSVRSCVLAKNGVRLRVATKGPC
agrin_caeel_fs2 VCSSHGVDYQSSCHLRHHACESKT...NITVKFFGRC
agrin_apime_fs1 VCGSDNVTYTNYCHLRKSSCLERK...STRVKNQGAC
agrin_apime_fs2 VCGTDGTDYPSLCEMNRAACAKGA...NITMAFQGKC
agrin_strpu_fs1 VCGSDGTTYLSECFLDKASCEQKK...RVYVASQGSC
agrin_strpu_fs2 ICGSDGVSYGNTCEMEAASCRQQK...EITLVNEGMC
agrin_cioin_fs1 ICGSDGVTYPNECRLRAAECMAQH...RIKIKATTSC
agrin_cioin_fs2 VCGSDQRSHRSSCDLRRASCVEGR...VITVVRNSAC
agrin_chick_fs1 VCGSDYSTYSNECELEKAQCNQQR...RIKVISKGPC
agrin_chick_fs2 VCGSDGKDYRSECDLNKHACDKQE...NVFKKFDGAC
agrin_rat_fs1   VCGSDASTYSNECELQRAQCNQQR...RIRLLRQGPC
agrin_rat_fs2   VCGSDGVDYPSECQLLSHACASQE...HIFKKFNGPC
```
