# Supplementary material for: Agrin Binds BMP2, BMP4 and TGFβ1
Source: PLoS One. 2010 May 21;5(5):e10758. doi: 10.1371/journal.pone.0010758 (PMC2874008; doi:10.1371/journal.pone.0010758)
Supplement: Figure S3 — Multiple alignment of EGF-domains of agrins. The abbreviations are: agrin_triad_egf1, agrin_triad_egf2, agrin_triad_egf3, agrin_triad_egf4 - EGF domains of the agrin of Trichoplax adhaerens; agrin_strpu_egf1, agrin_strpu_egf2, agrin_strpu_egf3 - EGF domains of the agrin of Strongylocentrotus purpuratus; agrin_cioin_egf1, agrin_cioin_egf2, agrin_cioin_egf3 - EGF domains of the agrin of Ciona intestinalis; agrin_rat_egf1, agrin_rat_egf2, agrin_rat_egf3, agrin_rat_egf4 - EGF domains of the agrin of Rattus norvegicus. (0.01 MB PDF) [file pone.0010758.s004.pdf]

```
agrin_triad_egf1 C...RTFRCFQNGQC...LNRTFGYYTCQCPLGYTGTYC
agrin_triad_egf2 C...SPNPCQNGGGC.SPLSG.NRYFQCACPTDYAGLLC
agrin_triad_egf3 C...ASNPCPANSTC.THEAGKSDSWKCLCPLGKIGQRC
agrin_triad_egf4 CQR...KPCQNGAQC..SLGVQG..YLCNCPQYYTGENC
agrin_strpu_egf1 C...DSSPCQHGGTC.QNDEI.APGFRCICPLGKGGPVC
agrin_strpu_egf2 C.QSKEMPCFNNGLC.EALNAES..YRCICQGDFFGTLC
agrin_strpu_egf3 C...EGHMCHEESTC.VALPE.GG.YRCDCPDGRMGDMC
agrin_cioin_egf1 CI...PNPCQHSANCFITMQANIFTNKCECKDNYEGETC
agrin_cioin_egf2 C...SPNPCQGGAKC.IEMPG.EEEFTCKCPPGRSGSLC
agrin_cioin_egf3 CYR...NPCDNGGVC..HPRGAE..YMCVCLPYYTGDNC
agrin_rat_egf1   C...DSQPCLHGGTC.QDQDS.GKGFTCSCTAGRGGSVC
agrin_rat_egf2   C...LPNPCHGGALC.QALEA.GM.FLCQCPPGRFGPTC
agrin_rat_egf3   C...QPNPCHGAAPC.RVLSS.GG.AKCECPLGRSGTFC
agrin_rat_egf4   CTQALGNPCLNGGSC..VPREAT..YECLCPGGFSGLHC
```
