# Supplementary material for: Agrin Binds BMP2, BMP4 and TGFβ1
Source: PLoS One. 2010 May 21;5(5):e10758. doi: 10.1371/journal.pone.0010758 (PMC2874008; doi:10.1371/journal.pone.0010758)
Supplement: Figure S4 — Multiple alignment of laminin G domains of agrins. The abbreviations are: agrin_triad_lamg1, agrin_triad_lamg2, agrin_triad_lamg3, agrin_triad_lamg4, agrin_triad_lamg5, agrin_triad_lamg6 - Laminin G domains of the agrin of Trichoplax adhaerens; agrin_apime_lamg1, agrin_apime_lamg2, agrin_apime_lamg3 - laminin G domains of the agrin of Apis mellifera; agrin_strpu_lamg1, agrin_strpu_lamg2, agrin_strpu_lamg3 - laminin G domains of the agrin of Strongylocentrotus purpuratus; agrin_human_lamg1, agrin_human_lamg2, agrin_human_lamg3 - laminin G domains of the agrin of Homo sapiens. (0.04 MB PDF) [file pone.0010758.s005.pdf]

```
agrin_triad_lamg1 AIPSFHDGSYIRFKNTSTWDR....EVNLTIEFMPSVFDGLILIGGNLVSS
agrin_triad_lamg2 FSPYFGGQSYLEYTD.KTSDYFAASSVTINLKVKPLKPNGLIFYGSQREDN
agrin_triad_lamg3 PSVAFDGGNVYTLRNNISADEKEFNFNTIELQLKTFNLHGLILWSGPRNYS
agrin_triad_lamg4 PSLAFDGRSAYPYLNNVDNRLTVSSIQSFQLSFKTLSSNGLILWHG.....
agrin_triad_lamg5 PAYWFTPTSYITLLSAVTEKKNSFSSNRYEFLVRGYH.NGLLLWLGQSQPN
agrin_triad_lamg6 PPVSFSGSKAVRYLSDVNRGLRAFRQNSLRLTFKTRKSTAVLFWLG..NDN
agrin_apime_lamg1 EVASFDGKSYVRMNRLKAY.....HKFSVEVEFKTYADNGIILYNQQKSDG
agrin_apime_lamg2 LIPEMSGDGFLELPCLEGVAKA....FSIELWFLTHASDGLLLYNGQLNNG
agrin_apime_lamg3 RPVRFKGDNFLQFRHRNGRRRKGQQSNKFELRLRTTHPDGLIAWIG.....
agrin_strpu_lamg1 TTPSFAGDSYLAYPEMDAF.....MEVEIVIEFQPGATEGVLLYEAQTAEG
agrin_strpu_lamg2 VVPGFAGNSYMQLPSLM.MPDD....SVIDVEFLTSSPDGVIFYNGQTADG
agrin_strpu_lamg3 DPLRFDGQTSIQYYNGVSKKQRALRTHQIQLSFKTAEPNGALFWNG..VGN
agrin_human_lamg1 PVPAFEGRSFLAFPTLRAY.....HTLRLALEFRALEPQGLLLYNG.NARG
agrin_human_lamg2 FLADFNGFSHLELRGLHTFARDLGEKMALEVVFLARGPSGLLLYNGQKTDG
agrin_human_lamg3 DTLAFDGRTFVEYLNAVTESEKALQSNHFELSLRTEATQGLVLWSGKATER

agrin_triad_lamg1 ....NGDYFALSLRSRYIE.........ASFELGSGKGTA.SSKIAVEVNR
agrin_triad_lamg2 ....RGDFILLNLVEGYLEFR.........FDLGSGT.AVIRSASPLTLNN
agrin_triad_lamg3 QNQR.RPFIALAVTNGNIQLR.........FNLGTQDNHIT.STVRINDGN
agrin_triad_lamg4 .NQKPGDFISVGIRNKFVELN.........YNLGSGTKTLTGGWQQVNDDK
agrin_triad_lamg5 PNR...DYLGVAIVNNRVQLR.........FNVGSGKIEIVVDVI.VTDGY
agrin_triad_lamg6 DPRLRSDYMSAAVEAGYFKIR.........YNLGGGATSSTILTLRVDDNQ
agrin_apime_lamg1 ....TGDFVSLAIVDGHVQFR.........YNLGNG.PVILTSPERVTMKT
agrin_apime_lamg2 ....RGDFISLNLVQAKLEFR.........FNLGSGI.ANITSPDPVTLDT
agrin_apime_lamg3 ..RGKVEHLILSLHGGQVLLTYKSKNEQIS..........LRSRERVDDGV
agrin_strpu_lamg1 ....NGDFISLAIVNNQVEFRFLATDQSNGFDLGSAEPVVITSTVDLQLMT
agrin_strpu_lamg2 ....RGDFISLNMRDGYLEFR.........YDLGSSI.AEIKSVDRLALNE
agrin_strpu_lamg3 .....ADFQAVGVSDGYVEYA.........YNLGRGITRI.RTTQKVDNNK
agrin_human_lamg1 ....K.DFLALALLDGRVQLR.........FDTGSG.PAVLTSAVPVEPGQ
agrin_human_lamg2 ....KGDFVSLALRDRRLEFR.........YDLGKGA.AVIRSREPVTLGA
agrin_human_lamg3 .....ADYVALAIVDGHLQLS.........YNLGSQPVVL.RSTVPVNTNR

agrin_triad_lamg1 WYRATVKRSGRDFTIQVNEEPPVVGKVPGSLSELNSLTDIYLGTLPSGLST
agrin_triad_lamg2 SHDINITRNGRYGTMRIDQQPEVRGIASGSFVLLSLFAPFYFGGH..P.NF
agrin_triad_lamg3 WHTIKAYRIGRQANLTVDTEF.RSTTVSTGSPQIAVDGDLFLGGLNTTPKK
agrin_triad_lamg4 WHKVLVDRQSRYGNLTVDGRS.ITGFANPSATELNAADYLYLGGVENIAAK
agrin_triad_lamg5 WHKVKIERNGSSGTLTVDGVSQKSN.PREGASVLNVDGNILIGGTPNIATT
agrin_triad_lamg6 WHTVYINRNLTNALLYVD.HLETSAISLQFQQELNVASYFYLGGVPE....
agrin_apime_lamg1 FHSVAAKRYHKDGVLIFNDGEDVVGQSQGMLKSLDLNQDTFVG.NMPT.NY
agrin_apime_lamg2 WHCVRISRLGREGVLQLDDGTVARGLSGSPLTELNLEMPLYVGGL..K.HW
agrin_apime_lamg3 FHQIRASRRRRTSMIQVDDSAPVKVSTE..MTLLTTNGKLFVGGKPG....
agrin_strpu_lamg1 WHRLRAYRSRREGSLSVDGEPEVTGTSEGVSGALNLGEDLFIGYAVPP.E.
agrin_strpu_lamg2 WHAVRVIRMGKSGEMILNDLPPVKGTSPPGASQLNLRQPLFIGGV..R.SY
agrin_strpu_lamg3 WHTVIITRNLIDASLQVDNEEPVMGQSRAGASQLDTDGFLYLGQGVNVP..
agrin_human_lamg1 WHRLELSRHWRRGTLSVDGETPVLGESPSGTDGLNLDTDLFVG.GVPE.DQ
agrin_human_lamg2 WTRVSLERNGRKGALRVGDGPRVLGESPVPHTVLNLKEPLYVGGA..P.DF
agrin_human_lamg3 WLRVVAHREQREGSLQVGNEAPVTGSSPLGATQLDTDGALWLGGLPELPVG

agrin_triad_lamg1 SNQFRSRIQMNQNFGGCIRSLDINNIEYNLNVPSSNKPDNVVSYYQLTRCV
agrin_triad_lamg2 AAMN.SKTKIKTGLVGCIESVTINGQEKQL......VKD.AIYGAGVSSCN
agrin_triad_lamg3 NW..NL...YRYNYVGCIRNIQIQHHKVNI......VTDLKVPQ.QHTTCE
agrin_triad_lamg4 TS..NL...YYSFYEGCVKEVKLSGVSLNM......QRDIQIRQ.RLYTCQ
agrin_triad_lamg5 TS..NK...YLENYRGCLKNFVIDGFVVNL......QL.MATESVNTKSCP
agrin_triad_lamg6 NNIDYT...SNDNFIGCMANVEVNGKIVQL......SDDAVDPNISLPRCQ
agrin_apime_lamg1 SKV.YENIGTNHGFLGCIRKLKINRIHVDLHV...GRDKEILETYRVKECG
agrin_apime_lamg2 REIH.RLAGARTGLVGAIQRLMVNGKTYQN......LA.VNVTQHNTEIYD
agrin_apime_lamg3 ..........HRGIKGCVSDFVVDKRRLQLGRR............RIEYCH
agrin_strpu_lamg1 ..VGLRLANTNQGFVGCIRYVEINSQELDISS...S.GSSVEYGANVGECG
agrin_strpu_lamg2 GEVS.RRAAITDGLNGAVRRFVVNEVDYST......LKDFAEAKVNVEEFR
agrin_strpu_lamg3 GSMESS...Y.TYYTGCIQDVLLDEVPLHL......YENAQGEKPSLF.CS
agrin_human_lamg1 AAVALERTFVGAGLRGCIRLLDVNNQRLELGI...G.PGAATRGSGVGECG
agrin_human_lamg2 SKLA.RAAAVSSGFDGAIQLVSLGGRQLLT......PEHV.LRQVDVTSFA
agrin_human_lamg3 PALPKA...YGTGFVGCLRDVVVGRHPLHL......LEDAV.TKPELRPCP
```
