# Supplementary material for: Agrin Binds BMP2, BMP4 and TGFβ1
Source: PLoS One. 2010 May 21;5(5):e10758. doi: 10.1371/journal.pone.0010758 (PMC2874008; doi:10.1371/journal.pone.0010758)
Supplement: Figure S5 — Multiple alignment of Laminin EGF-domains of agrins. The abbreviations are: agrin_caeel_lamegf1, agrin_caeel_lamegf2 - laminin EGF-domains of the agrin of Caenorhabditis elegans; agrin_apime_lamegf1, agrin_apime_lamegf2 - laminin EGF-domains of the agrin of Apis mellifera; agrin_strpu_lamegf1, agrin_strpu_lamegf2 - laminin EGF-domains of the agrin of Strongylocentrotus purpuratus; agrin_cioin_lamegf1, agrin_cioin_lamegf2 - laminin EGF-domains of the agrin of Ciona intestinalis; agrin_chick_lamegf1, agrin_chick_lamegf2 - laminin EGF-domains of the agrin of Gallus gallus; agrin_rat_lamegf1, agrin_rat_lamegf2- laminin EGF-domains of the agrin of Rattus norvegicus. (0.01 MB PDF) [file pone.0010758.s006.pdf]

```
agrin_caeel_lamegf1  CQCNRVGSFGHTCD.ETGQCKCRPGVAGIKCDHCLPSFWGIH.LIAQGALSC
agrin_caeel_lamegf2  CGCSAFGSSRSDCEQTTGKCECKNGALGDKCDLC.PNG......SMMTAGGC
agrin_apime_lamegf1  CNCNRLGSVSDTCNPETGQCECKPGVGGLKCDRCMPGYWGLPKI.SEGHQGC
agrin_apime_lamegf2  CGCSLFGSVREDCEQMTGRCVCKPDIQGQKCTICTDHN......KILTLTGC
agrin_strpu_lamegf1  CNCNPLGSYSVYCEPSNLQCPCKPGVGGKQCDRCEPGYYDFLSLERNG.IGC
agrin_strpu_lamegf2  CGCSSYGSRRDDCDQTSGACRCRRKAIGLKCNMC.PEG......LTMTPMGC
agrin_cioin_lamegf1  CLCNEHGSYGNACNPTTGQCVCRPGVGGLRCDRCRPGYWNFRALAEKLFTGC
agrin_cioin_lamegf2  CRCHEFGSTRDDCDQMTGRCSCKVGVTGLKCTDCVSDG......SWVTSSVC
agrin_chick_lamegf1  CQCNPYGSYGGTCDPATGQCSCKPGVGGLKCDRCEPGFWNFRGIVTDSKSGC
agrin_chick_lamegf2  CNCDPVGSVRDDCEQMTGLCSCKTGITGMKCNQC.PNG......SKMGMAGC
agrin_rat_lamegf1    CHCNPHGSYSGTCDPATGQCSCRPGVGGLRCDRCEPGFWNFRGIVTDGHSGC
agrin_rat_lamegf2    CSCDPRGAVRDDCEQMTGLCSCRPGVAGPKCGQC.PDG......QVLGHLGC
```
