# Supplementary material for: Agrin Binds BMP2, BMP4 and TGFβ1
Source: PLoS One. 2010 May 21;5(5):e10758. doi: 10.1371/journal.pone.0010758 (PMC2874008; doi:10.1371/journal.pone.0010758)
Supplement: Figure S6 — Multiple alignment of SEA domains of agrins. The abbreviations are: agrin_cioin_sea - SEA domain of the agrin of Ciona intestinalis, agrin_disom_sea - SEA domain of the agrin of Discopyge ommata; agrin_danre_sea - agrin of the SEA domain of Danio rerio; agrin_chick_sea - SEA domain of the agrin of Gallus gallus; agrin_human_sea - SEA domain of the agrin of Homo sapiens. (0.01 MB PDF) [file pone.0010758.s007.pdf]

```
agrin_cioin_sea AAVQFIGQGTFYSIDGNSLRHSKSLDVPGSEEFINYSNLVEA
agrin_disom_sea PTKLFQGVLIVEEVEGQELFYTPEMDDPKSELFGETARSIEN
agrin_danre_sea STMRFSGFLHLDKVEGQEVFYTPEMEDPKSELFGETARSIES
agrin_chick_sea ATKVFQGVLILEEVEGQELFYTPEMADPKSELFGETARSIES
agrin_human_sea ATKVFQGVLELEGVEGQELFYTPEMADPKSELFGETARSIES

agrin_cioin_sea EIMSLISNPPLLDSVRAVRISSFRSASIFWGGVIVTFELHL.
agrin_disom_sea ALNELFGNSNVKKDFKSVRVHGLGPSD....PVRIIVEVHFD
agrin_danre_sea AMNELFRKSDVQKDFQSVHVRNLSPSN....SIIAHIEAHFD
agrin_chick_sea ALDELFRNSDVKNDFKSIRVRDLGQSS....AVRVIVESHFD
agrin_human_sea TLDDLFRNSDVKKDFRSVRLRDLGPGK....SVRAIVDVHFD

agrin_cioin_sea .TSGSDAGAIQDAL..NAASSENRYFTISELTDAERMLEEYT
agrin_disom_sea PRTSYNSHDVQRALLQQVKQSRRKSIVVKKPEQDNVKIVDFD
agrin_danre_sea PDTRFNVGDIEGALLKQLKASKDTGIVVKKPEEENIHINNYG
agrin_chick_sea PATSYTAADVQAASLKQIRASKKRTILVKKPQQEHVKFMDFD
agrin_human_sea PTTAFRAPDVARALLRQIQVSRRRSLGVRRPLQEHVRFMDFD
```
