# Supplementary material for: Agrin Binds BMP2, BMP4 and TGFβ1
Source: PLoS One. 2010 May 21;5(5):e10758. doi: 10.1371/journal.pone.0010758 (PMC2874008; doi:10.1371/journal.pone.0010758)
Supplement: Figure S7 — Multiple alignment showing a region of the second LamG domain affected by alternative splicing in vertebrate agrins: the A/y splice site (see Figure 1). The abbreviations are: agrin_triad - agrin of Trichoplax adhaerens; agrin_caeel - the agrin of Caenorhabditis elegans; agrin_caebr - the agrin of Caenorhabditis briggsae; agrin_apime - the agrin of Apis mellifera; agrin_trica - the agrin of Tribolium castaneum; agrin_strpu - the agrin of Strongylocentrotus purpuratus; agrin_cioin - the agrin of Ciona intestinalis; agrin_disom - the agrin of Discopyge ommata; agrin_chick - the agrin of Gallus gallus; agrin_rat - the agrin of Rattus norvegicus; agrin_human - the agrin of Homo sapiens. Note that vertebrate agrins contain a conserved four-residue insert, KSRK, at the A/y splice site (positions underlined); analysis of genomic sequences revealed that this motif is missing in invertebrate agrins. (0.02 MB PDF) [file pone.0010758.s008.pdf]

```
agrin_triad LKVKPLKPNGLIFYGSQREDNRGDFILLNLVEGYLEFRFDLGSGTAVIRS
agrin_caeel IVFKPYRTNGILFYWSVPSDPHTDFIAFAMIDAKPHFVYELGSGLSYIRG
agrin_caebr IAF.PYRTNGLLFYWSVPSDPHTDFIAFAMIDAKPHFVYELGSGLSYIRG
agrin_apime LWFLTHASDGLLLYNGQLNNGRGDFISLNLVQAKLEFRFNLGSGIANITS
agrin_trica VYFMPKAANGLILYNGQLKNGRGDFISLNLARGHLQFRFNLGSGIANLTT
agrin_strpu VEFLTSSPDGVIFYNGQTADGRGDFISLNMRDGYLEFRYDLGSSIAEIKS
agrin_cioin ILFYSNQPDGLIFYNGQKKSGKGDFVSLNLKNGFLEFKYNLGQGAANIRS
agrin_disom VIFLAKDPNGMIFYNGQKTDGRGDFVSLNLRDGYLEFKYDLGKGAAVLRS
agrin_chick VVFLAKSPSGMIFYNGQKTDGKGDFVSLALHDGYLEYRYDLGKGAAVLRS
agrin_rat   MVFLARGPSGLLLYNGQKTDGKGDFVSLALHNRHLEFCYDLGKGAAVIRS
agrin_human VVFLARGPSGLLLYNGQKTDGKGDFVSLALRDRRLEFRYDLGKGAAVIRS

agrin_triad ASPLTLNNSHDINITRNGRYGTMRIDQQPEVRGIAS...GS.FVLLSLFA
agrin_caeel .EPIPLNSWHTVRIERLAKDVSMFV.NETLVKKHTS.QSKNAHLDISKKD
agrin_caebr .EPIPLNSWHTVRIERFAKDVSMYV.NGTLAKKYTS.QSKNAHLDILKND
agrin_apime PDPVTLDTWHCVRISRLGREGVLQLDDGTVARGLSG....SPLTELNLEM
agrin_trica KETVNIGKWHWARIFRDGREGILQLDNSSIVRGYSG....TPLTELNLEL
agrin_strpu VDRLALNEWHAVRVIRMGKSGEMILNDLPPVKGTSP....PGASQLNLRQ
agrin_cioin ANPVSLNEWHIVVLSRAMRTGDLSLDNFDPVYGTSP....SQHSFLDLKQ
agrin_disom KAPIPLNVWNVVTVERNGRKGLMKINKDELVSGESPKSRKAPHTALNLKE
agrin_chick KEPVPLNTWISVLLERSGRKGVMRINNGERVMGESPKSRKVPHAFLNLKE
agrin_rat   KEPIALGTWVRVFLERNGRKGALQVGDGPRVLGESPKSRKVPHTMLNLKE
agrin_human REPVTLGAWTRVSLERNGRKGALRVGDGPRVLGESPKSRKVPHTVLNLKE

agrin_triad PFYFGGHPNFAAMNSKTKIKTGLVGCIESVTI
agrin_caeel ALYVGFVPEGIISHKVRKLNVPFEGELQELRI
agrin_caebr VLFVGFVPNGEISHKVRKLNVPFEGELQELRI
agrin_apime PLYVGGLKHWREIHRLAGARTGLVGAIQRLMV
agrin_trica PFYIGSLSEWDEVHRLSGASKGYKGVIQRILL
agrin_strpu PLFIGGVRSYGEVSRRAAITDGLNGAVRRFVV
agrin_cioin PMYVGGFPDGVKFNPEAGVTTGLSGALQKFQV
agrin_disom AFYVGGAPDFNKFARAAGIISGFTGAIQKLSL
agrin_chick PFYVGGAPDFSKLARAAAISTSFYGAVQRISI
agrin_rat   PLYIGGAPDFSKLARGAAVSSGFSGVIQLVSL
agrin_human PLYVGGAPDFSKLARAAAVSSGFDGAIQLVSL
```
