# Supplementary material for: Agrin Binds BMP2, BMP4 and TGFβ1
Source: PLoS One. 2010 May 21;5(5):e10758. doi: 10.1371/journal.pone.0010758 (PMC2874008; doi:10.1371/journal.pone.0010758)
Supplement: Figure S8 — Multiple alignment of a region affected by alternative splicing and neurotrypsin cleavage in vertebrate agrins: the B/z splice site and β neurotrypsin cleavage site (see Figure 1). The alignment encompasses the C-terminal part of the fourth EGF domain and the N-terminal part of the third LamG domain. The abbreviations are: agrin_triad - agrin of Trichoplax adhaerens; agrin_trica - the agrin of Tribolium castaneum; agrin_apime - the agrin of Apis mellifera; agrin_cioin - the agrin of Ciona intestinalis; agrin_disom - the agrin of Discopyge ommata; agrin_chick - the agrin of Gallus gallus; agrin_rat - the agrin of Rattus norvegicus; agrin_human - the agrin of Homo sapiens. Note that vertebrate agrins contain a conserved eight-residue insert, xLxNEIPx, at the B/z splice site (positions underlined); analysis of genomic sequences revealed that this motif is missing in invertebrate agrins. The alignment also includes the β neurotrypsin cleavage site (arrow) of vertebrate agrins (see Figure 1). Note that in vertebrate agrins the β neurotrypsin cleavage site is conserved (positions double-underlined); analysis of genomic sequences revealed that this motif is missing in invertebrate agrins. (1.09 MB PDF) [file pone.0010758.s009.pdf]

```
                                                 β site
                                                   ↓
agrin_triad  CQNGAQCSLGVQGYLCNCPQYYTGENCSQAI......EVFGPSVAFDGGNVYTLRNNISA
agrin_trica  CLNNGLCVPELDQFICKCPSNTKGKYCE......ISQEETP.AIKFNGATFLQFRN....
agrin_apime  CHNGGVCLPLLNSYLCKCATGYNGLHCEFFMGYDVSTELTERPVRFKGDNFLQFRH....
agrin_cioin  CDNGGVCHPRGAEYMCVCLPYYTGDNCEQEHSTDLLQDEQATAIYLDGTTKIMYRNAVKA
agrin_disom  CQNGGVCSPRLREYDCMCQRGFSGPQCEKALEEKSASGSESVA..FNGRTFIEYHNTVTR
agrin_chick  CQNGGTCSPRLESYECACQRGFSGAHCEKVIIEKAAGDAEAIA..FDGRTYMEYHNAVTK
agrin_rat    CLNGGSCVPREATYECLCPGGFSGLHCEKGLVEKSVGDLETLA..FDGRTYIEYLNAVIE
agrin_human  CLNGASCVPREAAYVCLCPGGFSGPHCEKGLVEKSAGDVDTLA..FDGRTFVEYLNAVTE

agrin_triad  ........DEKEFNFNTIELQLKTFNLHGLILWSGPRNYSQNQRRPFIALAVTNGNIQL
agrin_trica  ....RGYRRRKPERGNRYEIKLRTFASDALLLWRSK...SRSIREDYFSIAVVNGYPEI
agrin_apime  ....RNGRRRKGQQSNKFELRLRTTHPDGLIAWIGR...G...KVEHLILSLHGGQVLL
agrin_cioin  I........SRARTHNNYEIVFRTTARHGLLLMVGK...ARE.GVDYIALAIHDGRLHL
agrin_disom  SHLANEIPDEKAVQVNYFEMSIKTEATKGLILWSGK...IAE.KSDYIALAVVDGFVQM
agrin_chick  SHLSNEIPAEKALQSNHFELSIKTEATQGLILWSGK...GLE.RSDYIALAIVDGFVQM
agrin_rat    SELTNEIPAEKALQSNHFELSLRTEATQGLVLWIGK...AAE.RADYMALAIVDGHLQL
agrin_human  SELTNEIPVEKALQSNHFELSLRTEATQGLVLWSGK...ATE.RADYVALAIVDGHLQL
```
