# Supplementary material for: Agrin Binds BMP2, BMP4 and TGFβ1
Source: PLoS One. 2010 May 21;5(5):e10758. doi: 10.1371/journal.pone.0010758 (PMC2874008; doi:10.1371/journal.pone.0010758)
Supplement: Figure S9 — Multiple alignment of regions affected by neurotrypsin cleavage (arrow) in vertebrate agrins: the α site (see Figure 1). The abbreviations are: agrin_disom - the agrin of Discopyge ommata; agrin_danre - the agrin of Danio rerio; agrin_chick - the agrin of Gallus gallus; agrin_rat - the agrin of Rattus norvegicus; agrin_human - the agrin of Homo sapiens. Note that in vertebrate agrins the α neurotrypsin cleavage site is conserved (positions double-underlined); analysis of genomic sequences revealed that this motif is missing in invertebrate agrins. (0.48 MB PDF) [file pone.0010758.s010.pdf]

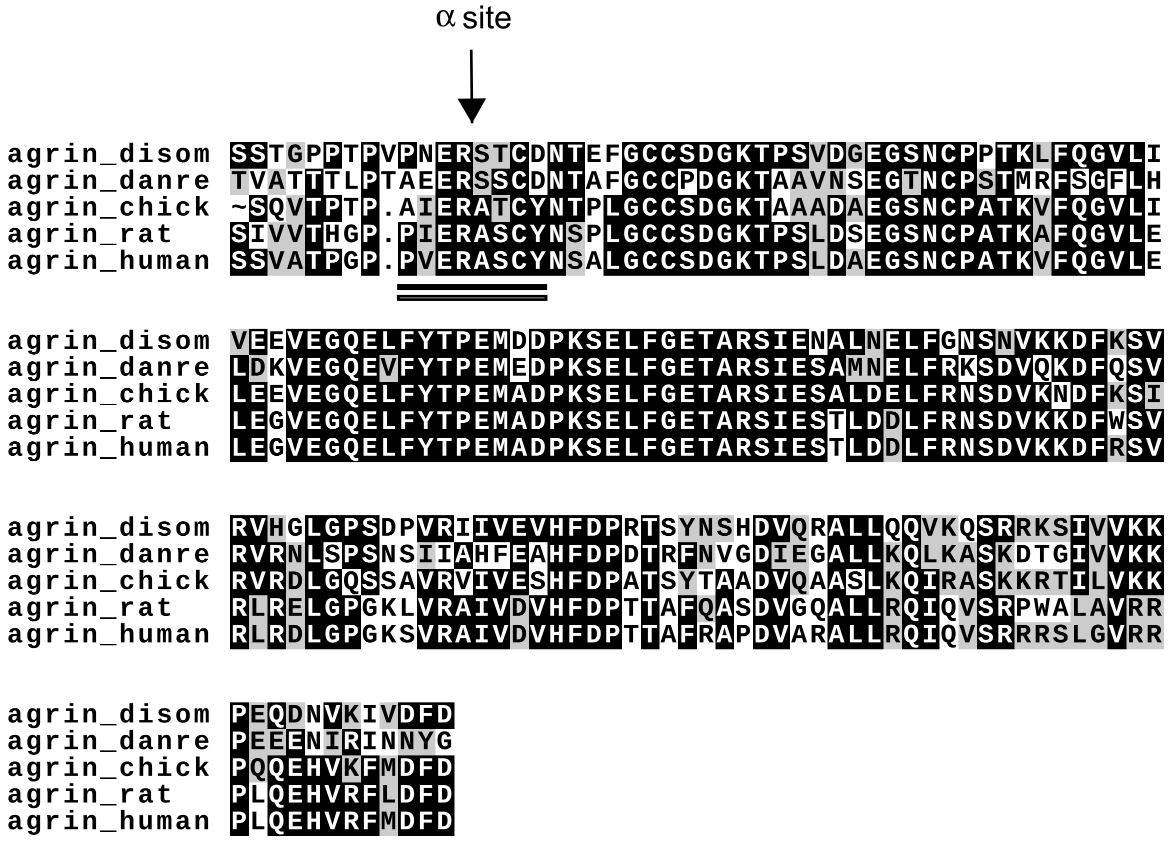
α site
agrin_disom SSTGPPTPVPNERSTCDNTEFGCCSDGKTPSVDGEGSNCPPTKLFQGVLI
agrin_danre TVATTTLPTAEERSSCDNTAFGCCPDGKTAAVNSEGTNCPSTMRFSGFLH
agrin_chick ~SQVTPTP.AIERATCYNTPLGCCSDGKTAAADAEGSNCPATKVFQGVLI
agrin_rat SIVVTHGP.PIERASCYNSPLGCCSDGKTPSLDSEGSNCPATKAFQGVLE
agrin_human SSVATPGP.PVERASCYNSALGCCSDGKTPSLDAEGSNCPATKVFQGVLE
agrin_disom VEEVEGQELFYTPEMDDPKSELFGETARSIENALNELFGNSNVKKDFKSV
agrin_danre LDKVEGQEVFYTPEMEDPKSELFGETARSIESAMNELFRKSDVQKDFQSV
agrin_chick LEEVEGQELFYTPEMADPKSELFGETARSIESALDELFRNSDVKNDFKSI
agrin_rat LEGVEGQELFYTPEMADPKSELFGETARSIESTLDDLFRNSDVKKDFWSV
agrin_human LEGVEGQELFYTPEMADPKSELFGETARSIESTLDDLFRNSDVKKDFRSV
agrin_disom RVHGLGPSDPVRIIVEVHFDPRTSYNSHDVQRALLQQVKQSRRKSIVVKK
agrin_danre RVRNLSPSNSIIAHFEAHFDPDTRFNVGDIEGALLKQLKASKDTGIVVKK
agrin_chick RVRDLGQSSAVRVIVESHFDPATSYTAADVQAASLKQIRASKKRTILVKK
agrin_rat RLRELGPGKLVRAIVDVHFDPTTAFQASDVGQALLRQIQVSRPWALAVRR
agrin_human RLRDLGPGKSVRAIVDVHFDPTTAFRAPDVARALLRQIQVSRRRSLGVRR
agrin_disom PEQDNVKIVDFD
agrin_danre PEEENIRINNYG
agrin_chick PQQEHVKFMDFD
agrin_rat PLQEHVRFLDFD
agrin_human PLQEHVRFMDFD
